# Maternal Epigenetic Signatures Linking Prenatal Serum PFAS Concentrations to Birth Size in an African American Maternal–Child Cohort

**DOI:** 10.1021/acs.est.6c03113

**Published:** 2026-09-23

**Authors:** Youran Tan, Anne L. Dunlop, Todd M. Everson, Alicia K. Smith, Stephanie M. Eick, Ziyin Tang, Shudi Pan, Neha Sehgal, Dana Boyd Barr, Kurunthachalam Kannan, P. Barry Ryan, Carmen J. Marsit, Donghai Liang

**Affiliations:** † Gangarosa Department of Environmental Health, Rollins School of Public Health, 1371 Emory University, Atlanta, Georgia 30322, United States; ‡ Department of Gynecology and Obstetrics, School of Medicine, Emory University, Atlanta, Georgia 30322, United States; § Wadsworth Center, 1094 New York State Department of Health, Albany, New York 12237, United States

**Keywords:** PFAS, Epigenomics, Mixtures, Birth outcomes

## Abstract

Per- and polyfluoroalkyl substance (PFAS) exposures have been linked to reduced birth size, but the underlying epigenetic mechanisms remain unclear. Among 296 mother–infant dyads in the Atlanta African American Maternal–Child Cohort (2016–2020), early pregnancy maternal blood samples (8–14 weeks) were processed for serum PFAS assessment and blood mononuclear cell (PBMC) DNA methylation profiling. We assessed birth size using sex-specific birth weight *z*-scores (BWZ) and small-for-gestational-age (SGA) status. Individual and mixture PFAS methylation associations were estimated by using robust generalized linear regression and quantile g-computation. We also conducted differentially methylated region (DMR), gene ontology, sex-specific, and exploratory mediation analyses. Overall, 132 CpGs were associated with at least one PFAS (FDR < 0.05), including 34, 60, 32, and 10 linked with PFHxS, PFOS, PFOA, and PFNA, respectively. PFOS showed the largest effects and appeared to drive the mixture associations. Six CpGs (annotated to *EVA1A, FCRL1, CTIF, ABHD12, and WDYHV1*) were associated with PFAS levels and birth size indicators. We identified 2–9 DMRs associated with individual PFASs and their mixture. Sex-specific PFAS methylation associations were observed. The PFAS-associated genes are involved in the inflammatory response, immune activity, and lipid homeostasis. Prenatal serum PFAS concentrations were associated with alterations in maternal DNA methylation, which in turn were associated with infant birth size.

## Graphical abstract

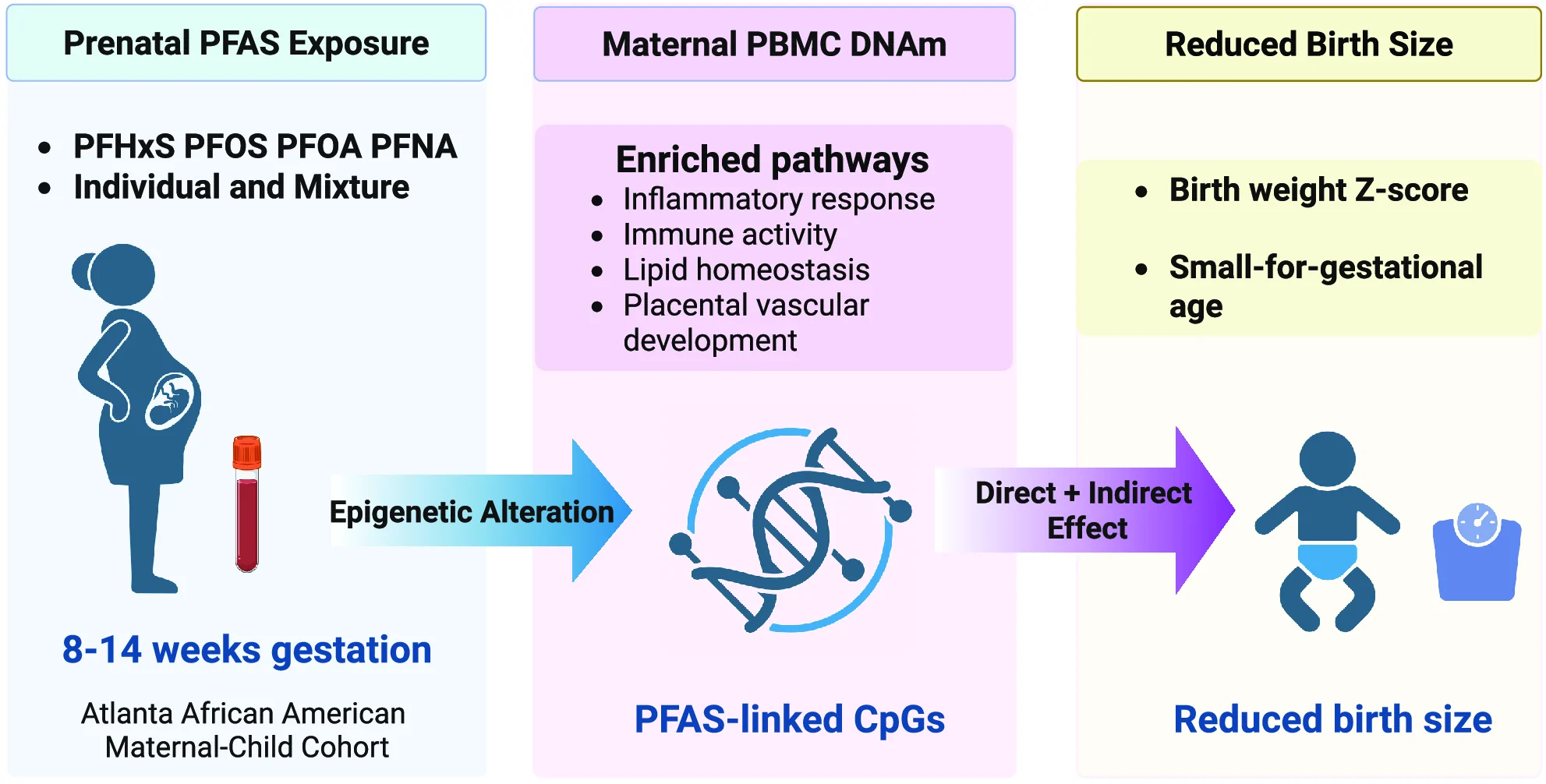

## Introduction

Per- and polyfluoroalkyl substances (PFAS) are a group of synthetic chemicals widely used in industrial processes and consumer products such as nonstick cookware, food packaging, water-repellent textiles, and firefighting foams.^1^ Due to their environmental persistence and long biological half-lives, PFAS are ubiquitous and can bioaccumulate in the human body.^2^ This widespread exposure is of particular concern during pregnancy, as PFAS can readily cross the placenta, leading to fetal exposure. Importantly, beyond direct fetal transfer, PFAS may also disrupt maternal physiology and placental function, including immune regulation, endocrine signaling, and metabolic processes.^3,4^ Alterations in maternal and placental biology during critical windows of pregnancy may, in turn, have downstream consequences for fetal growth and development. In particular, numerous epidemiological studies have linked maternal PFAS concentrations to adverse pregnancy and birth outcomes, including preeclampsia, preterm birth, reduced fetal growth, and developmental delay.^5^ However, the molecular mechanisms underlying PFAS-induced pregnancy and developmental toxicity still need to be elucidated to identify the early intervention opportunities.

In epidemiologic research, birth weight for gestational age is often used as an indirect measure of fetal growth, since true assessment requires serial ultrasound measurements across gestation, which are often unavailable, particularly in uncomplicated pregnancies.^6^ Consequently, small-for-gestational-age (SGA), defined as a birth weight below the 10th percentile for gestational age, is commonly used as a postnatal proxy for reduced fetal growth.^7^ Importantly, African American (AA) populations experience a disproportionately higher risk of adverse birth outcomes such as preterm birth, low birth weight, and reduced fetal growth compared to other racial groups.^8^

One potential mechanism through which prenatal PFAS exposure influences later health outcomes is via epigenetic modifications, particularly DNA methylation,^9^ which can persist through cell division and influence gene expression. PFAS could conjugate with intracellular glutathione (GSH), reducing cellular S-adenosylmethionine (SAM) levels and leading to hypomethylation of genome-wide DNA.^10^ Prenatal PFAS concentrations have been linked to global methylation of DNA and altered methylation of insulin-like growth factor 2­(*IGF2*) in cord blood,^11^ implicating epigenetic dysregulation in fetal development. Moreover, PFAS may influence birth size through disruptions in the growth hormone (GH)/insulin-like growth factor (IGF) axis, a key endocrine pathway regulating fetal growth.^12^ Thus, PFAS-induced DNA methylation changes could be a potential molecular mechanism linking PFAS exposure to adverse developmental and health outcomes.

Recent molecular epidemiological studies have examined the associations between prenatal PFAS concentrations and epigenetic changes during pregnancy and across various developmental stages, including in neonates, children, and adolescents, primarily in cord blood,^13–15^ peripheral leukocytes,^16^ placenta,^10,17–19^ and newborn dried blood spots.^20^ However, existing research remains limited in several ways. Most studies have examined only individual PFAS compounds without considering potential mixture effects, even though PFAS typically co-occur in real-world exposure scenarios. Moreover, few have comprehensively evaluated the associations between multiple PFAS concentrations, DNA methylation patterns, and birth outcomes such as birth size.^10,21^ Notably, none of these studies have specifically focused on minority populations. In addition, nearly all prior studies have focused on fetal or placental tissues, while maternal DNA methylation changes, which may capture early biological responses to PFAS exposure and potentially influence fetal development, remain largely unexplored.

To address these knowledge gaps, we performed an epigenome-wide association study (EWAS) to investigate the associations between individual and mixture PFAS concentrations and maternal DNA methylation profiles in maternal blood during early pregnancy. This study was conducted within the prospective Atlanta African American Maternal–Child cohort, a socioeconomically diverse population. Additionally, we examined whether the identified differentially methylated CpG sites (CpGs) were associated with birth size indicators, providing insight into potential epigenetic mechanisms linking prenatal PFAS concentration to fetal growth.

## Method

### Study Population

This study leveraged data from a subset of participants enrolled in the Atlanta African American Maternal–Child Cohort, a well-established prospective birth cohort that has been described thoroughly elsewhere.^22,23^ Briefly, pregnant participants were recruited from prenatal care clinics affiliated with Emory Midtown Hospital (privately funded) and Grady Hospital (publicly funded). Eligibility criteria included presenting for their first prenatal clinic visit between 8 and 14 weeks of gestation with a singleton pregnancy, a maternal age between 18 and 40 years, self-identifying as African American or Black, being able to communicate in English, and having no diagnosis of chronic medical conditions or chronic use of a prescription medication.

In this ongoing cohort, 710 participants have been enrolled to date. For the current study, we retrieved data from 510 participants enrolled between 2014 and 2020 who had available prenatal serum PFAS measurements and offspring birth outcome data. PFAS measurements were available for participants enrolled before 2020. The exposure and birth size analyses were conducted in this larger analytic sample of 510 participants to maximize the statistical power. Among these participants, maternal DNA methylation data were available for 296 participants. Therefore, all downstream methylation-related analyses were conducted among the 296 participants. We also repeated the exposure and birth size analyses within the methylation subset (*N* = 296) as a sensitivity analysis to evaluate whether the associations observed in the larger analytic cohort were comparable among participants included in the epigenetic analyses. The detailed participant selection process and full analytic workflow are presented in Figure S1. All participants provided informed consent for enrollment. This study was reviewed and approved by the Institutional Review Board of Emory University (IRB no. 68441).

### Exposure Assessment

Maternal venous blood samples were collected during early pregnancy (8–14 weeks) and allowed to clot. Clotted samples were centrifuged, processed to collect serum, and then stored at −80 °C until further analysis. PFAS were analyzed at two laboratories participating in the Children’s Health Exposure Analysis Resource (CHEAR) program, the Wadsworth Center and the Laboratory of Exposure Assessment and Development for Environmental Research (LEADER) at Emory University. CHEAR was an exposure assessment resource, and the laboratories have implemented the same quality control procedures for harmonized data.^24^ Briefly, each serum sample was spiked with isotopically labeled internal standards, extracted by solid-phase extraction, and quantified by liquid chromatography–tandem mass spectrometry (LC-MS/MS). Isotope dilution calibration was used to quantify PFAS concentrations. Analytical QA/QC procedures included matrix-based calibration curves, batch blanks, quality control samples, and standard reference materials. For the LEADER method, each analytical batch included a matrix-based calibration curve, a blank sample, and quality control samples analyzed alongside unknown serum samples. Limits of detection (LODs) ranged from 0.02 to 0.20 ng/mL and were defined as the lowest calibration concentration with a signal-to-noise ratio ≥ 3 and accuracy within 100 ± 20%. Method precision, calculated as relative standard deviation, was <10% for all analytes. National Institute of Standards and Technology standard reference materials were analyzed concurrently, and recoveries of target analytes were within 100 ± 20%. For the Wadsworth method, serum PFAS were extracted using hybrid solid-phase extraction and quantified using ultraperformance liquid chromatography–tandem mass spectrometry. Method validation showed intraday recovery of 88.7–117%, intraday precision of 1.0–13.4%, interday precision of 2.8–6.9%, and limits of quantification of 0.05–0.09 ng/mL. The Wadsworth method was also validated using standard reference materials and external proficiency testing samples. Further details of the analytical methods for PFAS quantification have been described elsewhere.^25,26^ Four PFAS, including perfluorohexanesulfonic acid (PFHxS), perfluorooctanesulfonic acid (PFOS), perfluorooctanoic acid (PFOA), and perfluorononanoic acid (PFNA), were measured at both laboratories, detected in >90% of participants, and included in the current study. PFAS concentrations below the LOD were imputed with LOD/√2.^27^

### DNA Methylation

As part of routine clinical procedures during early pregnancy (8–14 weeks), an additional 12 mL of maternal peripheral venous blood was collected using the same needle stick and drawn into an EDTA-containing tube. Blood was transferred into SepMate tubes with a Ficoll density gradient to isolate peripheral blood mononuclear cells (PBMCs) from whole blood. PBMCs were stored in AllProtect Buffer (Qiagen, Catalog # 76405) at −80 °C until a simultaneous DNA extraction using the AllPrep DNA Mini Kit (Qiagen, Catalog # 80204, Venlo, Netherlands) was performed according to the manufacturer’s instructions. DNA quantification and quality were assessed using the Quant-it Pico Green kit (Invitrogen, Catalog# P11496, Carlsbad, California).^28^

The MethylationEPIC version 1 BeadChip (Illumina) was used to assess DNA methylation at 850 k CpG sites quantitatively across the genome. Briefly, 1 μg of DNA was processed and hybridized to the BeadChip, according to the manufacturer’s instructions. Initial data quality control was performed using the R package CpGassoc.^29^ Any CpG site with a low signal or missing data for greater than 10% of samples was removed, and any sample with missing data for greater than 5% of CpG sites was removed. Cross-reactive probes,^30^ and CpGs on chromosomes *X* and *Y* were excluded. As a result, 8,01,158 CpG probes were included in the working set. β-mixture quantile normalization (BMIQ) was performed as previously described.^31^ Briefly, BMIQ involves fitting a three-state β-mixture model, transforming the distribution of type 2 probes to match the type 1 distribution, followed by a dilation transformation.^31^ We performed all statistical analyses using logit-transformed *M*-values = $\log_{2}\left(\frac{\beta }{1-\beta }\right)$ , where β is the ratio of intensities measured by methylated probes and the sum of intensities measured by both methylated and unmethylated probes.^32^

To account for heterogeneity in PBMC composition across participants, estimated cell type proportions (CD8+ T cells, CD4+ T cells, natural killer cells, B cells, and monocytes) were included as covariates in the analysis. These estimates were derived using a reference-based deconvolution method, based on the data set developed by Reinius et al.^33^ and implemented following the approach described by Houseman et al.^34,35^

### Birth Size and Other Maternal Factors

Birth size was characterized using two measures: birth weight for gestational age *z*-score (BWZ) as the continuous outcome and SGA, defined as <10th percentile for gestational age and infant sex, as the categorical outcome, using the population information from the U.S. natality files for singleton births in 2017.^36^ Gestational age at delivery was determined from the delivery record using the best obstetrical estimate based on the date of delivery in relation to the estimated date of confinement (EDC) established by the last menstrual period and/or early ultrasound.^37^ Birth weight was determined from the first weight measured in the delivery room.

Clinical data were collected using a standardized chart abstraction tool to ascertain prenatal characteristics, including parity and early pregnancy body mass index (BMI). The sociodemographic survey was assessed using maternal self-report and prenatal administrative record reviews, including maternal age at study entry, marital status, educational attainment, type of health insurance, income-to-poverty ratio, and substance use (alcohol, tobacco, and marijuana) one month prior to the enrollment visit.

### Statistical Analysis

Descriptive analyses were performed for serum PFAS concentrations, including detection frequencies, geometric means (GMs), geometric standard deviations (GSDs), and distribution percentiles. Data were not normally distributed, thus all PFAS concentrations were naturally log-transformed to more closely approximate normality for model assumptions and reduce the potential effects of outliers.

We examined the associations between individual PFAS and BWZ and SGA by fitting multivariable linear regressions and logistic regressions, respectively. Considering the simultaneous exposure to multiple PFAS and high correlations between analytes, we further conducted quantile g-computation^38^ to explore the mixture effect of PFASs. To evaluate dose–response relationships, we used categorical individual PFAS concentration groups divided into quartiles to model BWZ and log odds of SGA.

#### Epigenome-Wide Association Study (EWAS)

We performed epigenome-wide association studies (EWAS) using robust generalized linear regressions to estimate the associations between individual PFASs and DNA methylation levels (*M*-values) at each of 8,01,158 CpGs. Models were adjusted for potential confounders and precision variables identified by a directed acyclic graph (Figure S2), including maternal age (years), gestational age at sample collection (weeks), infant sex, substance use (any vs none), BMI during early pregnancy, parity (any vs none), maternal education (categorical; less than high school, high school, some college, college and above), imputed cell type proportions, and surrogate variables (*sva* package in R), which was used to identify and adjust for other unwanted variation in the methylation data.^39^ In total, 24 surrogate variables were identified and controlled for in each individual EWAS model.

Next, we used quantile g-computation to explore the joint effect of a PFAS mixture on DNA methylation and the relative contribution of each PFAS within the mixture. We conducted a targeted mixture analysis on the CpGs that were significantly (false discovery rate (FDR) < 0.05) associated with any one of the PFAS congeners from individual PFAS EWAS. The primary output from a quantile g-computation model is the psi estimate, which represents the expected change in the *M*-values for a simultaneous one-quartile increase in all PFAS within the mixture, controlling for the same covariate set described above. Each PFAS in the mixture is assigned a positive or negative weight, reflecting the relative contribution of each exposure. Negative and positive weights sum to one.^38^

Raw *p*-values were adjusted for multiple comparisons using the Benjamini–Hochberg procedure, and FDR at 0.05 was used as a cutoff for significance. Bonferroni-adjusted *p*-values (<0.05) were additionally used to discuss higher confidence results. Genomic inflation was evaluated by constructing quantile–quantile (Q–Q) plots and calculating λ for each EWAS.

For each significant CpG (FDR *q* < 0.05), we conducted an exploratory follow-up analysis by assessing the associations with birth size indicators using robust generalized linear regression models. Models were adjusted for the same covariates as above, except for surrogate variables and infant sex, since SGA and BWZ accounted for infant sex. We reported both nominal *p*-values and FDR-adjusted *q*-values.

Finally, exploratory mediation analyses were conducted using the “mediation” package^40^ to evaluate whether CpGs associated with both PFAS and birth size indicators showed evidence consistent with a potential indirect pathway. Covariates were aligned with those included in the EWAS. We estimated the average causal mediation effect (ACME) but interpreted these estimates cautiously due to the cross-sectional nature of the study design. ACME *p*-values were therefore interpreted as exploratory evidence rather than confirmatory evidence of mediation. Nominal ACME *p*-values <0.05 were considered significant, and *p*-values between 0.05 and 0.10 were considered borderline significant.

#### Identification of Differentially Methylated Regions (DMRs)

DMRs were identified using the R package “*dmrff*”.^41^ CpGs with *p*-values< 0.05 from PFAS EWAS analysis were selected as seeds to detect potential DMRs. We specified the maximum distance to 1500 bp for features considered to be proximal consecutive CpGs for candidate DMRs. DMRs containing only a single assayed CpG were excluded. To identify the regional differential methylation among candidate regions, *dmrff* applies an extension of inverse-variance weighted meta-analysis to calculate a weighted average difference in DNA methylation for each candidate DMR. DMRs with Bonferroni-adjusted *p*-values < 0.05 were considered statistically significant.

To assess the associations between significant DMRs and birth size indicators, we first leveraged the regional association at these CpGs within each DMR and performed a principal component analysis (PCA). The first principal component (PC1), representing the major source of variation across CpGs within each DMR, was then tested for its association with the birth size indicators.

#### Gene Ontology (GO) Terms Enrichment Analysis

To improve the biological interpretation of PFAS-associated CpGs, we performed gene ontology (GO) enrichment analysis using “*methylglm*” in the “*methylGSA*” package,^42^ which utilizes the full list of raw *p*-values from the EWAS, making it independent of arbitrary significance thresholds and instead evaluating whether specific GO terms or pathways are overrepresented as the *p*-values get smaller. GO annotates genes to three biological domains: biological processes (BP), molecular functions (MF), and cellular components (CC). Since very few GO terms met the FDR *q* < 0.05, we applied a less stringent cutoff for the exploratory enrichment analysis. Accordingly, GO terms with FDR *q* < 0.2 were considered significant. Redundant GO term pathways were removed using REVIGO.^43^

#### Secondary Analysis

Emerging evidence suggests that endocrine-disrupting chemicals may exert sex-specific effects, and DNA methylation patterns differ by sex across both maternal and neonatal genomes, which could contribute to sex-biased gene expression and sexually differentiated traits.^44,45^ In the secondary analysis, we examined the potential effect modification by infant sex by including interaction terms in regression models for each PFAS. The interaction term was considered the primary statistical evidence for sex-specific associations. For the CpGs where effect estimates for both PFAS and the interaction term were significant (FDR *q* < 0.05), we further conducted sex-stratified analyses among mothers carrying female infants (*N* = 153) and male infants (*N* = 143) to describe the direction and magnitude of PFAS–methylation associations within each stratum. Since EWAS interaction analyses may be underpowered and unstable in modest sample sizes, these analyses were considered exploratory. We visualized the associations between PFAS and DNA methylation by infant sex, holding all other variables constant using the “*visreg*” package.

All statistical analyses were conducted with R version 4.5.1.

## Results

### Characteristics of the Study Population

Among the 296 participants included in the current analysis, the average age was 25 years (standard deviation [SD] = 4.67). More than half of the participants were multiparous (55%), had a high school education or less (55%), and had an income-to-poverty ratio less than 150% (70%). The average birth weight was 3030 g (SD = 585), and 38 (13%) infants were born with low birth weight (<2500 g), while 66 (22%) infants were SGA (Table 1). Overall, the methylation subset (*N* = 296) was broadly comparable to the larger analytic cohort (*N* = 510) for major maternal sociodemographic, clinical, and infant characteristics. No meaningful differences were observed between the methylation subset and the larger analytic cohort across the key characteristics evaluated (Table S1).

**Table 1:** Sociodemographic and Clinical Characteristics of Pregnant Women in the Atlanta African American Maternal–Child Cohort, 2014–2020 (*N* = 296)^a^

	*N* = 296
**maternal age**	
mean (SD)	25.0 (4.67)
**maternal early pregnancy BMI (kg/m** ^**2**^ **)**	
mean (SD)	28.4 (7.65)
**gestational age at delivery (weeks)**	
mean (SD)	38.4 (2.40)
**birth weight (grams)**	
mean (SD)	3030 (585)
**birth weight Z-score for gestational age**	
mean (SD)	–0.45 (1.07)
	***N* (%)**
**marital status**	
married or cohabiting	143 (48%)
not married or cohabiting	153 (52%)
**maternal education**	
<high school education	48 (16%)
high school or some college	116 (39%)
college degree	90 (30%)
graduate degree	42 (14%)
**income-to-poverty ratio**	
<100%	145 (49%)
100–150%	63 (21%)
150–300%	52 (18%)
>300%	36 (12%)
**delivery hospital**	
Emory	107 (36%)
Grady	189 (64%)
**parity**	
0	132 (45%)
1+	164 (55%)
**tobacco use prior to pregnancy**	
no	240 (81%)
yes	56 (19%)
**Marijuana use prior to pregnancy**	
no	188 (64%)
yes	108 (36%)
**alcohol use prior to pregnancy**	
no	265 (90%)
yes	31 (10%)
**infant sex**	
male	143 (48%)
female	153 (52%)
**LBW (<2500 g)**	
no	258 (87%)
yes	38 (13%)
**SGA (<10th percentiles)**	
no	230 (78%)
yes	66 (22%)

a Abbreviations: SD, standard deviation; BMI, body mass index; LBW, low birth weight; SGA, small-for-gestational-age; and N, sample size.

Maternal serum PFHxS (geometric mean [GM] = 0.94; geometric standard deviation [GSD] = 1.94 ng/mL), PFOS (GM = 1.87; GSD = 2.07 ng/mL), PFOA (GM= 0.62; GSD = 2.28 ng/mL), and PFNA (GM = 0.22; GSD = 2.36 ng/mL) were detected in >96% of the samples (Table S2). Spearman correlation coefficients between these four PFAS ranged from 0.34 to 0.76 (all *p*-values <0.05) (Table S3).

### Associations of Serum PFAS Concentrations with Birth-Size Indicators

As shown in Figure S3, each natural log-unit increase in PFNA concentration was associated with higher odds for SGA (odds ratio [OR] = 1.37 [95% CI: 1.04, 1.84]) and decreased BWZ (β = −0.11 [95% CI: −0.21, 0.00]) among the overall population (*N* = 510). Similarly, a simultaneous quartile increase in the PFAS mixture was significantly associated with higher odds of SGA (OR = 1.30 [95% CI: 1.00, 1.68]) and decreased BWZ (β = −0.1 [95% CI: −0.21, 0.00]). For the quartile analyses, the ORs of Q3 of PFOA and Q2 and Q4 of PFNA concentrations were significantly higher than Q1. We also observed a similar trend of increased odds of SGA and decreased BWZ with increasing individual PFAS and PFAS mixture among the current analytical study population (*N* = 296), but the results were not statistically significant (Figure S4).

### Epigenome-Wide Association Study (EWAS) of PFAS

After adjusting for potential confounders and multiple comparisons, 132 CpGs were significantly associated with at least one PFAS at FDR *q* < 0.05 (Figure 1A and Excel Tables S1–S4). We identified 34 significant CpGs for PFHxS, 60 for PFOS, 32 for PFOA, and 10 for PFNA at FDR *q* < 0.05 and 4 significant CpGs for PFHxS, 7 for PFOS, 4 for PFOA, and 1 for PFNA at Bonferroni *p* < 0.05 (Table S4). Three significant CpGs (FDR < 0.05) associated with both PFHxS and PFOS are cg14656695, located on *ABCA3* (chr16:2378802, effect size= 0.08 for PFHxS and 0.06 for PFOS), cg11709588, located on *RACGAP1* (chr12:50386759, effect size= −0.05 for PFHxS and PFOS), and cg17518710, located on *ZBTB16* (chr11:113954170, effect size= 0.09 for PFHxS and PFOS). Only one PFAS-associated CpG overlapped between PFOS and PFOA is cg13778564, located on *IPPK* (chr9:95376183, effect size = 0.09 for PFOS and 0.06 for PFOA). Q–Q plots suggested minimal overall inflation (inflation factor λ = 1.10 for PFHxS, 1.09 for PFOS and PFOA, and 1.03 for PFNA; Figure 1B).

**Figure 1:**
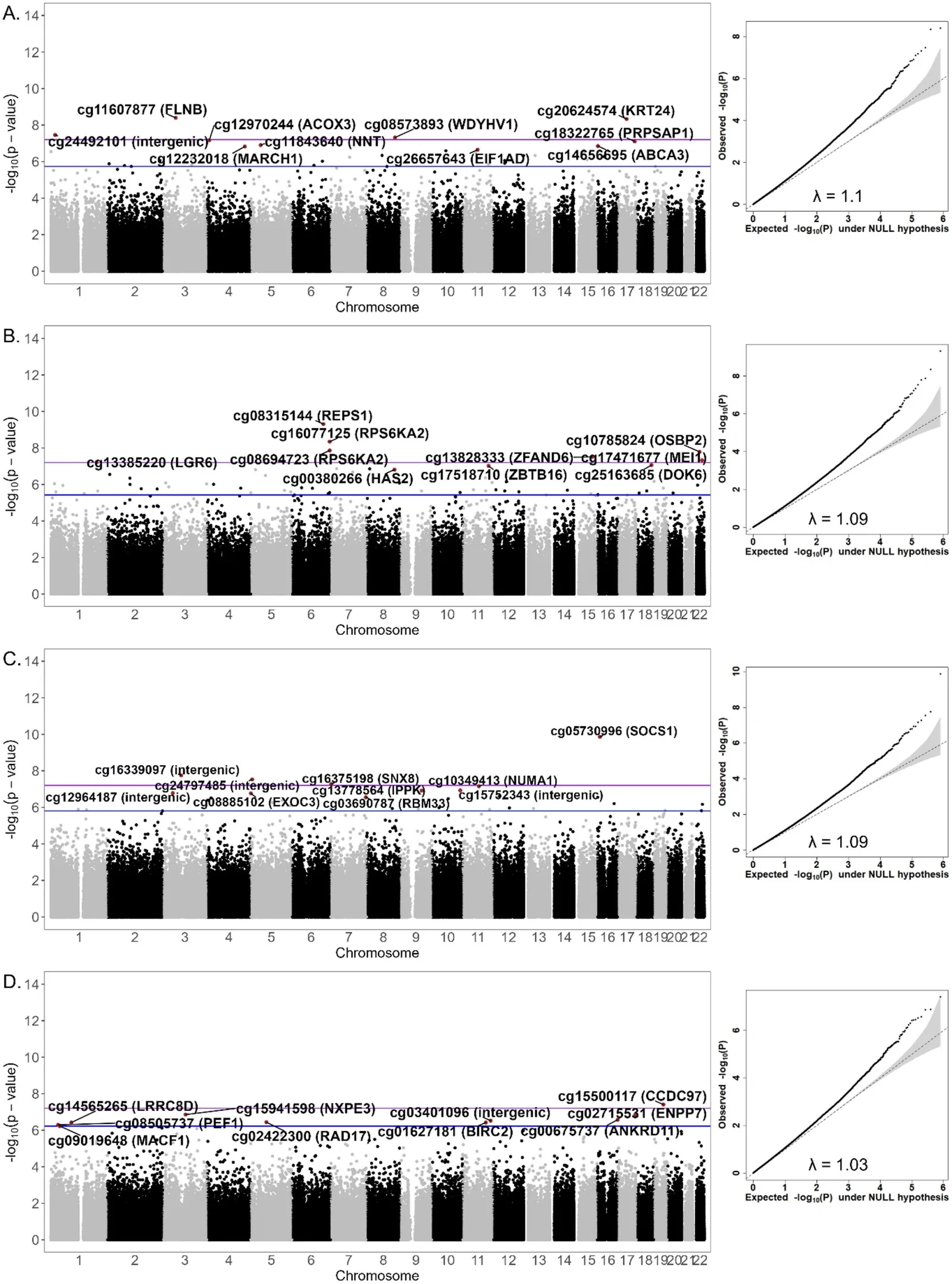
Manhattan plots and quantile–quantile (Q–Q) plots for the epigenome-wide associations of ln-transformed maternal serum (A) PFHxS, (B) PFOS, (C) PFOA, and (D) PFNA concentrations. Note: All models adjusted for maternal age, infant sex, maternal education, early pregnancy BMI, substance use, parity, gestational age at sample collection, cell types, and surrogate variables. In the Manhattan plots, the *x*-axis indicates genomic locations of CpGs, and the *y*-axis indicates −log_10_ (*P*-value) of the associations. The annotated dots represent the top ten significant CpGs with FDR *q* < 0.05. The upper line indicates the Bonferroni threshold <0.05, and the lower line indicates the threshold of FDR < 0.05. In the Q–Q plot, the *x*-axis and the *y*-axis show the expected −log_10_ (*P*-value) and the observed −log_10_ (*P*-value), respectively. Abbreviations: PFHxS, perfluorohexanesulfonate; PFNA, perfluorononanoate; PFOA, perfluorooctanoate; and PFOS, perfluorooctanesulfonate.

We further explored whether methylation of 132 differentially methylated CpGs identified above was significantly affected by simultaneous exposure to PFAS. Using quantile g-computation, we found that a simultaneous one-quartile increase in all four PFAS concentrations was associated (FDR *q* < 0.05) with hypomethylation at 47 CpGs and with hypermethylation at 51 CpGs (Figure 2 and Excel Table S5). Because of the large number of CpGs with significant associations with PFAS mixture, Bonferroni *p* < 0.05 are reported in the main text (total *n* = 17 CpGs, Figure 2). The top two CpGs are cg13778564, located on *IPPK* (chr9:95376183, psi = 0.06) and cg09096967, located on an intergenic region (chr3:128718681, psi = −0.04). The direction of the overall mixture effect aligned with the direction of the effects observed in individual PFAS models.

**Figure 2:**
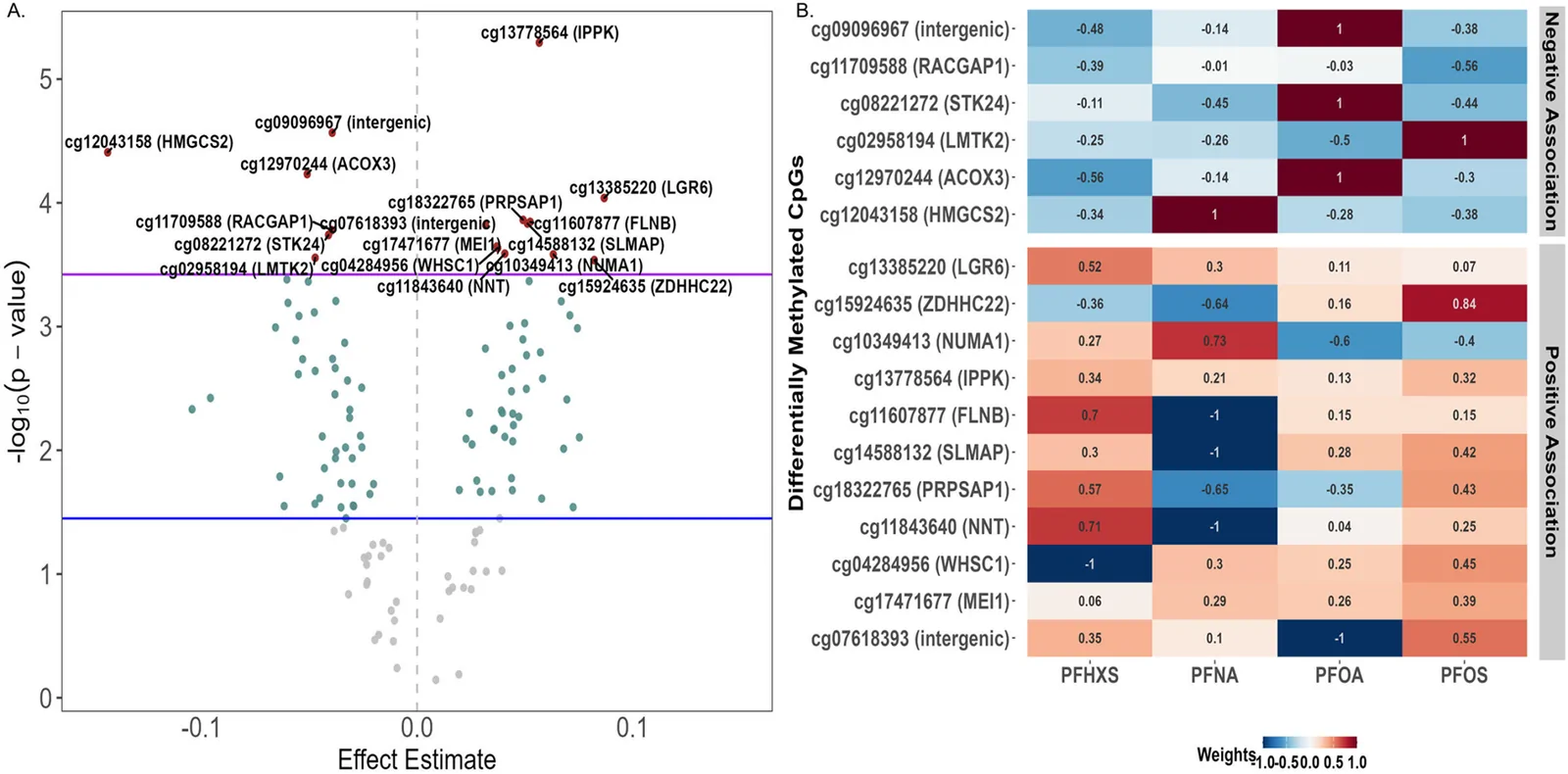
Epigenome-wide associations between per- and polyfluoroalkyl substances (PFAS) mixture and DNA methylation levels. (A) Volcano plots for the mixture effect on significant CpGs from individual models (FDR *q* < 0.05); (B) heatmap of positive and negative weights in the mixture showing the partial effects of each PFAS on DNA methylation levels of the significant CpGs from individual models (Bonferroni *q* < 0.05). Note: All models adjusted for maternal age, infant sex, maternal education, early pregnancy BMI, substance use, parity, gestational age at sample collection, cell types, and surrogate variables. In the volcano plot, the *x*-axis indicates the overall PFAS mixture effect, and the *y*-axis indicates −log_10_ (*P*-value) of the associations. The annotated dots represent significant CpGs with Bonferroni *q* < 0.05. The upper line indicates the Bonferroni threshold <0.05, and the lower line indicates the threshold of FDR < 0.05. In the heatmap, the *y*-axis is grouped based on whether the overall mixture effect of PFAS on DNA methylation levels was positive or negative. Within each row, the blue boxes show the negative weights and red-orange boxes show the positive weights. All negative weights in a row together sum to 1, and all positive weights in a row sum to 1. Abbreviations: PFHxS, perfluorohexanesulfonate; PFNA, perfluorononanoate; PFOA, perfluorooctanoate; and PFOS, perfluorooctanesulfonate.

Importantly, most PFAS compounds assigned the greatest weights in the mixture analysis also showed significant associations in the individual PFAS models (Excel Table S5). For overall negative mixture effects, PFHxS and PFOS always contributed to the greatest effect, whereas PFOA was often a nonfactor in the negative effects. Similarly, for overall positive mixture effects, PFHxS and PFOS were considered as main drivers, while PFNA often showed a minimal contribution. Overall, only 4 CpG sites (cg11709588, cg13385220, cg13778564, and cg17471677) with significant mixture effects exhibited the same direction of effects across all 4 PFASs.

### Differentially Methylated Regions (DMRs) for PFAS

For each individual PFAS and PFAS mixture, between two and nine DMRs containing at least two differentially methylated CpGs were identified (Excel Tables S6–S10). Most DMRs showed consistent hyper- or hypomethylation of CpGs within the region. Certain DMR-annotated genes were associated with multiple PFAS, such as *TTLL7* with PFOA and PFNA, and *SSBP3* with PFOS and PFOA. The most significant DMR (Bonferroni *q* = 4.37 × 10^–09^) was associated with PFHxS within the *METTL9* and *IGSF6* genes. The top DMR for PFAS mixture was annotated to *NPHS2* (chr6:179545458–179546285, included 4 CpGs in the region, Bonferroni *q* = 0.01).

### Gene Ontology (GO) Term Analysis

In the exploratory GO enrichment analysis, we observed significant enrichment of gene sets annotating to 29 unique GO terms using a less stringent FDR threshold of *q* < 0.2 (Figure 3), which allowed us to identify broader biological patterns while still controlling for multiple testing. Among them, 10 GO terms were significant for PFHxS, 7 for PFOS, 9 for PFOA, and 5 for PFNA. Acetyl-CoA metabolic process was a common hit for both PFOA and PFOS, and 3′-5- DNA helicase activity was a common hit for both PFHxS and PFOA. Several pathways, including insulin-like growth factor binding, 3′,5′-cyclic-AMP phosphodiesterase activity, and sulfation pathways, are particularly relevant to fetal growth and placental function. However, given the exploratory threshold used, these GO results should be interpreted as hypothesis-generating rather than definitive pathway-level evidence.

**Figure 3:**
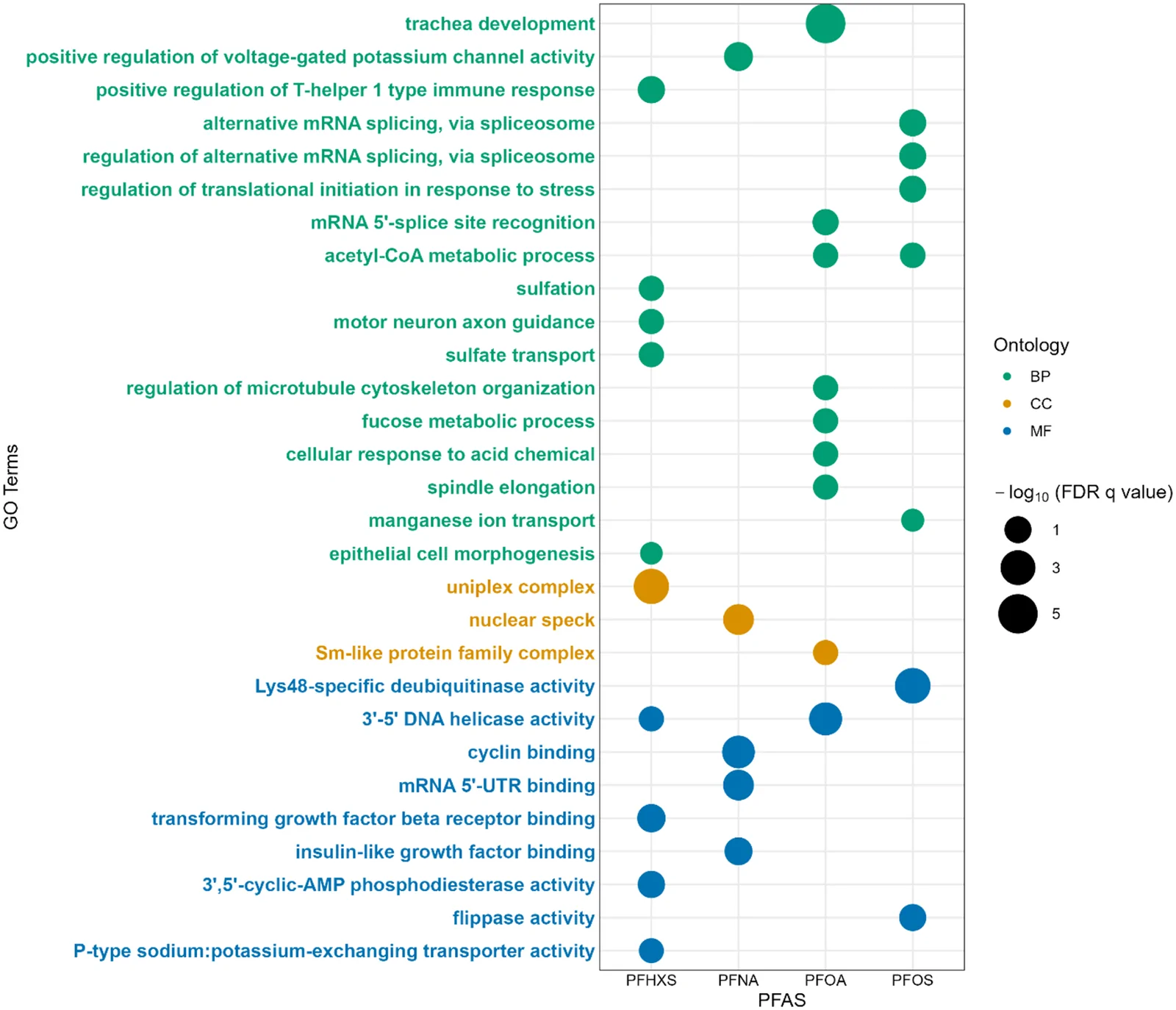
Gene ontology (GO) terms significantly enriched (FDR *q* < 0.2) in gene sets associated with maternal per- and polyfluoroalkyl substances (PFAS) concentrations. Abbreviations: BP, biological process; CC, cellular component; MF, molecular function; GO, Gene Ontology; PFHxS, perfluorohexanesulfonate; PFNA, perfluorononanoate; PFOA, perfluorooctanoate; and PFOS, perfluorooctanesulfonate.

### Associations of PFAS-Related CpGs/DMRs with Birth-Size Indicators

We examined the associations between 132 PFAS-related CpGs (FDR *q* < 0.05) and birth size indicators. Among these, methylation of 6 CpGs was suggestively associated (*p*-value <0.05) with SGA and BWZ (Figure 4 and Excel Table S11), and only two (PFOS-related cg24626310 annotated to *ABHD12*, and PFOA-related cg08933467 annotated to *FCRL1*) were nominally associated with both indicators. Two additional CpGs (PFOS-related cg26859549 annotated to *CTIF*, and PFOS-related cg01468161 annotated to intergenic region) were nominally associated with BWZ, and two other CpGs (PFHxS-related cg12838436 annotated to *EVA1A*, and PFHxS-related cg08573893 annotated to *WDYHV1*) with SGA. All six CpGs were significantly associated with the PFAS mixture (FDR *q* < 0.05). However, none of the CpG–birth size associations survived FDR correction at *q* < 0.05.

**Figure 4:**
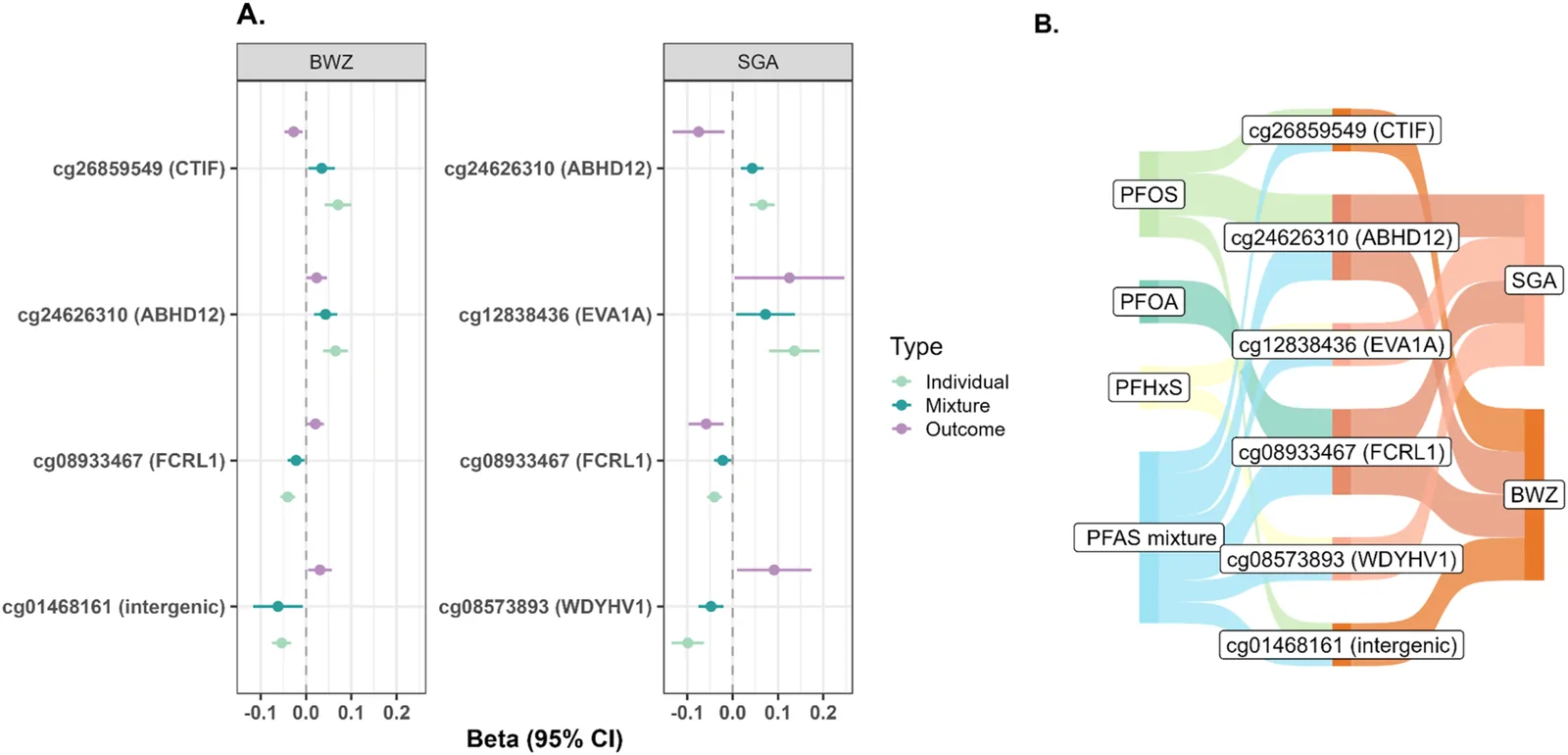
Associations between per- and polyfluoroalkyl substances (PFAS) related CpGs with birth size indicators. (A) Effect estimates for CpGs associated with individual PFAS, PFAS mixture (FDR *q* < 0.05), and birth size indicators (*p* < 0.05); (B) Sankey plot with the first node being exposures, leading to the associated CpGs in the second node, and ending at the birth size outcomes in the last node. Note: Exposure models were adjusted for maternal age, infant sex, maternal education, early pregnancy BMI, substance use, parity, gestational age at sample collection, cell types, and surrogate variables. Outcome models were adjusted for age, maternal education, early pregnancy BMI, substance use, parity, gestational age at sample collection, and cell types. Abbreviations: SGA, small-for-gestational-age; BWZ, birth weight z-score for gestational age; PFHxS, perfluorohexanesulfonate; PFNA, perfluorononanoate; PFOA, perfluorooctanoate; and PFOS, perfluorooctanesulfonate.

We conducted exploratory mediation analyses among the six PFAS (FDR *q* < 0.05) and birth size-related CpGs (*p*-value < 0.05). We found that 12.6% of the association between PFOS and BWZ (*p*-value = 0.02) may be mediated by cg26859549 *(CTIF)*. Cg08933467 *(FCRL1)* had a borderline significant negative indirect effect for the PFOA–BWZ association (*p*-value = 0.06) and a significant positive indirect effect for the PFOA–SGA association (*p*-value = 0.04) (Table S5).

For PFAS-related DMRs, SGA did not significantly account for the variance captured by the principal components of CpG methylation within these regions (Figure S5A). The first principal component (PC1) of PFAS-related DMRs was inversely associated with the risk of SGA and positively associated with BWZ (Figure S5B).

### Effect Modification by Infant Sex

We conducted the secondary analysis to examine whether each PFAS and the mixture’s associations with DNA methylation were differential among male and female infants, by including PFAS-by-sex interaction terms in the EWAS models. We observed sex-specific effects of PFHxS, PFOS, PFOA, and PFNA concentrations on 21, 43, 114, and 4 CpGs at FDR *q* < 0.05, respectively (Excel Tables S12–S15). For 19 of 178 unique CpGs, the main effects of PFAS were also significant, with FDR *q* < 0.05 (Figures 5, S6, and Excel Table S16). Sex-stratified estimates suggested that many CpGs had stronger PFAS–methylation associations among mothers carrying male infants than among those carrying female infants, indicating potential sex-specific epigenetic responses. PFOA showed the largest number of PFAS-by-sex interaction signals. Notably, PFHxS concentrations were associated with a significant increase in methylation levels in mothers carrying male infants at the *TNFRSF1B* gene, which encodes a receptor for tumor necrosis factor-α (TNF-α) and plays a critical role in regulating inflammatory responses.

**Figure 5:**
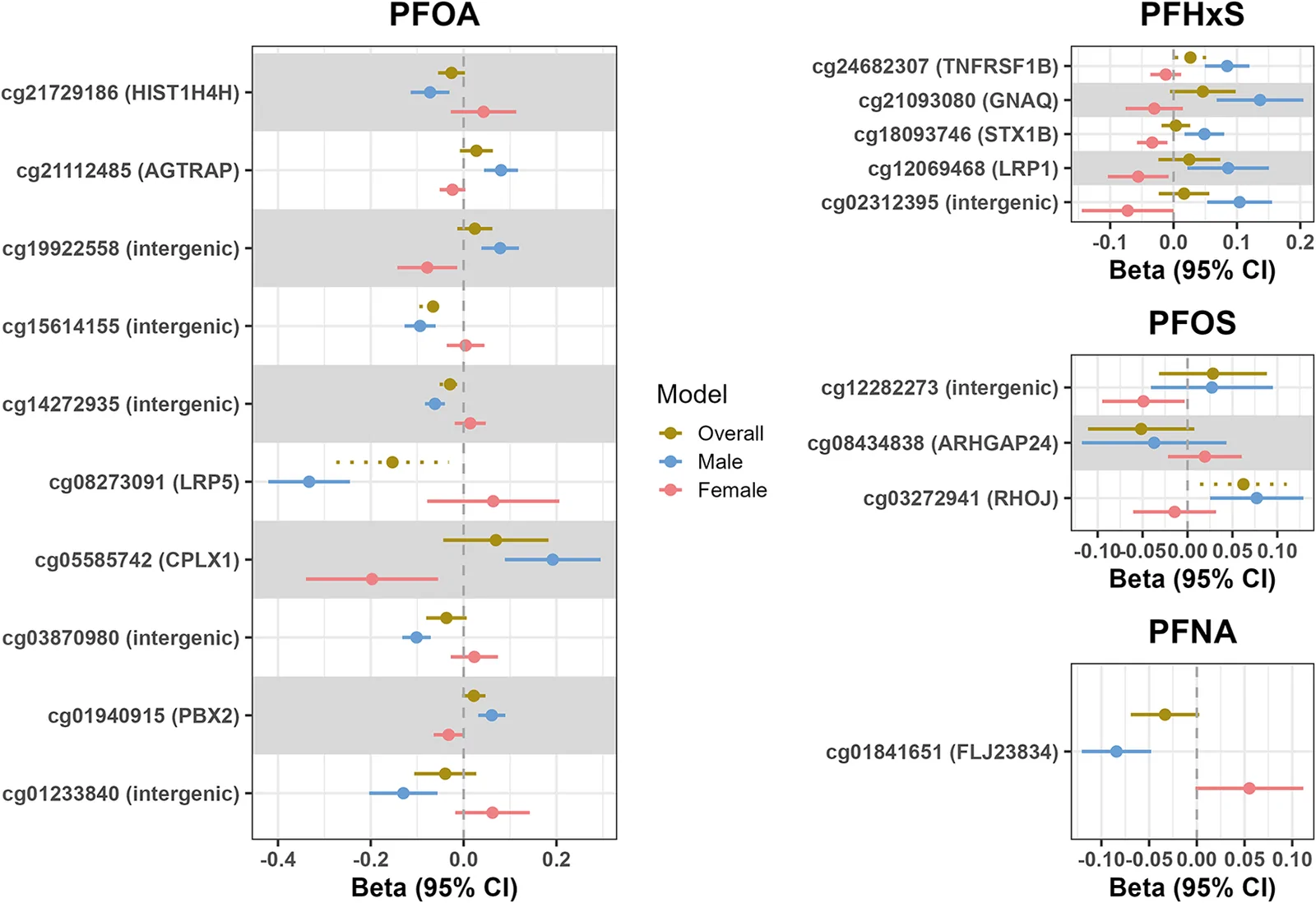
Forest plots showing the overall and sex-specific effects of per-and polyfluoroalkyl substances (PFAS) on DNA methylation levels of significant CpGs in the interaction models. Note: Only CpGs where the effect estimates of PFAS and the interaction term were significant (FDR *q* < 0.05) are shown. The dashed line represents the significant overall associations. Abbreviations: PFHxS, perfluorohexanesulfonic acid; PFOS, perfluorooctanesulfonic acid; PFOA, perfluorooctanoic acid; PFNA, perfluorononanoic acid; and CI, confidence interval.

## Discussion

In the first study of its kind, we investigated the associations between PFAS concentrations and DNA methylation in maternal blood and explored whether these variations in PFAS-linked DNA methylation are also associated with birth-size indicators. We identified 132 CpGs and 28 DMRs associated with the prenatal PFAS concentration. Additionally, six CpGs (annotated to *ABHD12*, *FCRL1*, *CTIF*, *EVA1A*, *WDYHV1*, and an intergenic region) were also associated with the birth size indicators. We also found evidence of sex-specific epigenetic associations with PFAS concentrations. Overall, these epigenetic changes have been involved in inflammatory response, immune activity, lipid homeostasis, and placental vascular development. To our knowledge, this is the first epigenome-wide association study of prenatal serum PFAS concentrations and birth size using maternal blood samples.

Previous EWASs conducted in birth cohorts have identified significant associations between prenatal PFAS concentrations and DNA methylation measured at birth or during early childhood. Several PFAS-associated CpGs from our study have also been identified in other studies using cord blood (Table S6). Specifically, nine CpGs we identified were also observed in a prospective health cohort of 583 mother–infant pairs from Colorado that examined maternal midpregnancy PFAS levels and cord blood DNA methylation.^13^ Genes annotated to these consistently identified PFAS-associated CpGs included *EPSTI1*, implicated in immune privilege and autoimmune diseases,^46^ and *PRPSAP1*, which is involved in nucleotide biosynthesis and plays a role in cell growth and proliferation.^47^ Starling et al. reported a negative association between prenatal PFOS concentrations and methylation at a CpG annotated to *SOCS1*, a negative regulator of cytokine signaling with a central role in modulating immune responses, inflammation, and trophoblast differentiation.^48^ Consistently, we also observed an inverse association of PFOA concentration and PFAS mixture concentration and methylation at this CpG (cg05730996). Notably, *SOCS1* has also been identified as a key predictor of the gestational age in the Norwegian Mother and Child Cohort Study (MoBa).^49^

In addition, five PFAS-associated CpGs that we identified have been previously linked to birth outcomes, particularly gestational length (Table S7). For example, DNA methylation in cord blood at CpGs annotated to *FGFBP2* was significantly associated with gestational age in two independent studies: one involving 1753 newborns from the MoBa cohort^49^ and another including 110 Japanese mother–infant pairs.^50^ *FGFBP2* encodes a fibroblast growth factor binding protein involved in modulating immune responses and inflammation through growth factor signaling pathways.^51^ Starling et al. and our study both noted significant negative associations between this site and PFHxS and PFOA, highlighting the potential role of *FGFBP2* in linking PFAS-related effects on gestational length.

A key novelty of the current study lies in its focus on maternal peripheral blood for epigenetic profiling during pregnancy. To date, nearly all existing birth cohort studies have utilized either cord blood or placental tissue for epigenetic analyses, with no prior studies conducted using maternal peripheral blood. Leveraging maternal blood offers unique insights into maternal physiological and immunological changes in response to prenatal PFAS levels and how these changes may influence fetal development through maternal-fetal interactions. Importantly, assessing DNA methylation in maternal samples enables the identification of exposure-related changes during early pregnancy, a critical window for fetal development and potential early intervention, while also capturing PFAS-related perturbations in maternal biological systems that play a central role in pregnancy success and overall gestational outcomes.

Interestingly, significantly differentially methylated CpGs in our study were annotated to genes implicated in key processes, such as inflammation. Specifically, PFHxS, PFOS, and the PFAS mixture concentrations were linked with methylation of CpGs annotated to *RACGAP1* and *ZBTB16*. *RACGAP1* plays a critical role in cytokinesis and has been linked to antiapoptotic activity, cell proliferation, and inflammation.^52^ Similarly, ZBTB16 is a transcriptional regulator of the NF-κB signaling pathway,^53^ a central mediator of inflammatory and immune responses. PFOS, PFOA, and PFAS mixture levels were associated with methylation changes at *IPPK*, a gene involved in the inositol phosphate pathway, which plays a role in immune signal transduction and chromatin regulation.^54^ Other genes with PFAS-associated differential methylation, including *RPS6KA2*, *MAPK1*, and *HAS2* (associated with PFOS concentrations), *BIRC2* (PFNA concentrations), *CD70* (PFHxS concentrations), and *LRRC20* (PFOA concentrations), were closely involved in inflammatory cytokine signaling, cellular stress responses, and immune modulation.^55–60^ Consistently, prior studies have linked PFAS to related molecular changes, including reduced *HAS2* expression among women undergoing assisted reproductive technology treatment,^61^ and differential *LRRC20* methylation among women exposed to high PFAS levels through contaminated drinking water.^62^ These gene-level findings were also consistent with the pathway analysis. We found several inflammation-related pathways, including positive regulation of T-helper 1 type immune response, regulation of translational initiation in response to stress, epithelial cell morphogenesis, transforming growth factor β receptor binding, and insulin-like growth factor binding. Among these pathways, insulin-like growth factor binding is also a key endocrine pathway regulating fetal growth, suggesting that PFAS-related epigenetic alterations may contribute to abnormal growth outcomes.^12^ Notably, a previous study in this cohort has shown strong associations between PFAS exposure and inflammatory biomarkers,^63^ and thus the current findings provide additional evidence supporting these associations. Together, our results align well with previous evidence linking PFAS to immune dysregulation, oxidative stress, and pro-inflammatory signaling,^64^ which may further affect placental function and subsequently influence infant growth and development.

Notably, we also found CpGs and DMRs associated with PFAS concentrations were annotated to genes involved in lipid metabolism and homeostasis. PFHxS, PFOS, and PFAS mixture were associated with methylation at CpGs annotated to *ABCA3*, a gene involved in phospholipid transport and surfactant phospholipid metabolism.^65^ Consistent with the biological relevance of this gene family, experimental evidence from differentiated HepaRG hepatocytes has shown that short-chain PFAS can alter the expression and activity of ABCA transporters, including *ABCA3*.^66^ Meanwhile, CpGs associated with PFHxS and PFAS mixture levels were also annotated to *ACOX3* and *NNT*. ACOX3 encodes a peroxisomal enzyme that catalyzes the β-oxidation of branched-chain fatty acids, thus maintaining cellular lipid homeostasis,^67^ while *NNT* encodes an antioxidant defense protein critical for mitochondrial redox homeostasis, lipid peroxidation, and energy balance.^68^ Consistent with these gene functions, prior mouse work reported that PFOA altered protein expression of immune mediators, including *NNT*.^69^ DMRs associated with PFHxS levels were also annotated to *INPP4A*, belonging to the family of lipid phosphatases that regulate phosphatidylinositol 3-kinase (PI3K) signaling and further affect critical cellular processes such as growth, proliferation, and apoptosis.^70^ Consistent with these gene-level findings, pathway analysis identified sulfation transport and the acetyl-CoA metabolic process, both of which are involved in steroid and lipid metabolism. Epidemiological evidence has demonstrated that PFAS are associated with perturbations in lipid metabolism among pregnant women,^71,72^ individuals undergoing laparoscopic bariatric surgery^73^ and young adults.^74^ Given that lipid metabolism during pregnancy is essential to provide fatty acids for fetal growth, the perturbation of lipids and fatty acids may contribute to the placental dysfunction and suboptimal fetal growth.^75,76^

Among the genes annotated to PFAS-associated DMRs, *PEG10* emerged as particularly compelling due to its essential role in fetal growth and placental development, especially in trophoblast cell function.^77^ Evidence from *PEG10* knockout mice demonstrates early embryonic lethality with severe placental defects, as well as fetal growth restriction beginning midgestation.^78^ In human studies, downregulated *PEG10* expression has been associated with spontaneous miscarriages,^79^ and has been observed in patients with early onset preeclampsia.^80^ In the present study, *PEG10* exhibited consistent hypermethylation across more than 60 PFHxS-associated CpGs, suggesting the potential disruption of placental function associated with PFAS concentrations. *RYR3*, also identified as a PFHxS-associated DMR, was hypermethylated within its genomic region. *RYR3* encodes a calcium channel expressed in trophoblast cells and regulates calcium-dependent cell migration, a process important for placental implantation and vascularization.^81^ Experimental evidence has also suggested that PFOS can disrupt calcium signaling genes like *RYR3* in zebrafish embryos.^82^ In the present study, however, methylation differences at these loci were observed in maternal PBMCs. Therefore, these findings may reflect maternal epigenetic signatures at genes with known relevance to pregnancy biology, but do not directly demonstrate altered placental methylation or placental dysfunction. Future studies with paired maternal blood, placenta, and cord blood data are needed to determine whether these maternal PBMC signatures correspond to molecular changes at the maternal-fetal interface.

PFAS developmental toxicity may involve multiple pathways, including peroxisome proliferator-activated receptors (PPARs) activation, which can disrupt lipid and glucose metabolism and placental functions.^3^ In addition, both experimental and epidemiological studies have suggested that PFAS may disrupt the growth hormone (GH)/insulin-like growth factor (IGF) axis, a major endocrine pathway controlling fetal growth, thereby contributing to abnormal fetal development.^12^ Consistent with this evidence, we observed that the insulin-like growth factor binding pathway was significantly associated with prenatal PFAS concentrations. *ETV6*, a gene involved in hematopoiesis and embryonic development,^83^ also showed a positive association with PFOS in our study, consistent with a prior study of blood samples from hospital-enrolled patients with diverse clinical conditions.^84^ This finding suggests a potential link between PFAS and developmental regulatory pathways.

In our study, six PFAS-associated CpGs showed nominal associations with birth size indicators. Among these, CpGs annotated to *FCRL1* and *ABHD12* were associated with both BWZ and SGA. Previous research in a Faroese birth cohort have reported n-PFOS were associated with CpG annotated to *FCRL1* among 72 cord blood samples.^14^ *FCRL1* encodes a B-cell receptor involved in B-cell activation, differentiation, and antibody production, supporting immune homeostasis during pregnancy.^85^ *ABHD12* encodes lyso-PS lipase that hydrolyzes bioactive lipids in the brain and immune cells among human.^86^ Other overlapping CpGs associated with both PFAS and birth size indicators were annotated to *EVA1A* and *CTIF*. *EVA1A* regulates autophagy and apoptosis and has been implicated in embryonic neurogenesis, cardiac remodeling, and islet α-cell function.^87^ In particular, *EVA1A* expression in the placenta has been reported to be upregulated in preeclampsia.^88^ *CTIF* encodes a translation initiation factor involved in mRNA surveillance and cellular stress adaptation.^89^ Together, our findings suggested that maternal DNA methylation patterns associated with prenatal PFAS exposure may also relate to infant birth size indicators, involving pathways related to lipid metabolism, immune regulation, and cellular stress response. Of note, while these biological functions are relevant to maternal immune and metabolic adaptation during pregnancy, the CpG–birth size findings should be interpreted as suggestive rather than robust downstream associations.

We observed the sex-specific associations between PFAS and maternal DNA methylation, which align with previous research. Toxicological evidence indicates that prenatal exposure to emerging PFAS compounds, such as F-53B, can impair longitudinal bone growth and induce osteoporosis in male offspring.^90^ Epidemiological studies, however, have reported inconsistent sex-specific findings, depending on the PFAS compound and outcome. For example, some studies found that effects of PFOA on head circumference and birth weight were stronger in female fetuses,^91,92^ whereas higher PFOS concentrations were associated with reduced birth size in male infants,^93^ while others reported no clear sex-dependent differences.^17,94^ Moreover, a growing body of epigenetic evidence has shown sex-specific effects of endocrine-disrupting chemicals, including PFASs, on cord blood and placental DNA methylation.^10,14,17,95^ Our study extends prior research by investigating the sex-specific effects of PFASs on maternal DNA methylation. PFAS are known to disrupt sex hormone synthesis and receptor activity, which could partly explain the observed sex-specific epigenetic response.^96^ Furthermore, fetal sex itself can influence maternal physiology, shaping glucose tolerance,^97^ immune milieu including cytokine levels,^98^ and metabolic response through pregnancy.^99^ These sex-dependent maternal adaptations could, in turn, modulate how maternal immune and metabolic pathways respond to PFAS concentrations at the epigenetic level.

To our knowledge, this is the first study to examine maternal epigenetic profiles linking individual and mixture PFAS with birth size. Maternal DNA methylation may provide early insight into exposure-related biological changes before alterations are detectable in the infant. In addition, we used Illumina MethylationEPIC BeadChip, which measures methylation at over 8,50,000 CpG sites and provides greater coverage of enhancer regions and distal regulatory elements than the Illumina HumanMethylation450 BeadChip.^100^ Importantly, our study population was exclusive to African Americans, who are often understudied and experience a disproportionately high risk of adverse birth outcomes. Our study focused on African American participants, an understudied population with a disproportionate burden of adverse birth outcomes. Of note, the mean BWZ of −0.45 in this cohort was below the national reference mean of 0, indicating that infants were, on average, smaller for gestational age and sex than the overall U.S. singleton reference population. This pattern is consistent with documented racial disparities in birth outcomes among AAs and may partly explain the elevated SGA prevalence of 22%. This context should be considered when interpreting the magnitude and generalizability of the PFAS and birth size associations.

Despite these promising findings, this analysis also has its own limitations. First, the associations between PFASs and DNA methylation were evaluated at a single time point. Thus, the observed relationships among PFAS exposure, DNA methylation, and birth size cannot be interpreted as causal. In addition, CpGs were selected for mediation analysis based on associations with both PFASs and birth size indicators, which may inflate mediation estimates. Therefore, these findings should be viewed as hypothesis-generating and require replication in longitudinal studies with repeated methylation profiling across pregnancy. Second, fetal growth was assessed using BWZ and SGA rather than longitudinal ultrasound-based growth measures, which were not available in this cohort. Although repeated ultrasonography is the gold standard for fetal growth trajectories, BWZ and SGA are clinically relevant birth-based indicators that capture cumulative in-utero growth restriction across gestation. Third, maternal PBMCs may capture PFAS-related maternal epigenetic responses, but placental or cord blood samples are more directly relevant to fetal development. Therefore, interpretations involving placental biology, vascular development, or fetal endocrine pathways should be considered to be inferential. Future studies with paired maternal blood, cord blood, placental tissue, and child biomarkers are needed to clarify these pathways. Still, we observed some consistency in epigenetic changes with prior studies using maternal PBMCs, which further underscores the value of early pregnancy maternal samples as an easily accessible biospecimen for early detection and intervention. Also, cell-type deconvolution used a reference panel from purified blood immune cells rather than a PBMC-specific panel. Although this panel captures major PBMC cell types, the estimated cell proportions may be imperfect. Meanwhile, PFAS mixture analysis should also be interpreted in the context of an analytic strategy. Although a full methylome-wide mixture analysis was first conducted, only one CpG passed the FDR correction. We thus focused on CpGs identified in individual PFAS EWASs to improve interpretability. Because this was a postselection approach, concordance between individual and mixture findings may be overestimated. Also, we cannot rule out the possibility of residual confounding by other factors, such as diet, nutritional status, stress, and lifestyle factors,^101^ as they can influence PFAS levels, one-carbon metabolism, inflammation, and immune regulation. Future studies with detailed dietary assessment, nutritional biomarkers, stress, and exposome-wide data are needed to assess the specificity of PFAS-related methylation and its relationship with the birth size. Lastly, replication of the current findings in larger and more diverse populations will also be essential to validate the observed associations and strengthen generalizability.

## Supplementary Material





## Notes

Top CpGs by *p*-value (FDR < 0.05) in adjusted robust linear regression models for maternal serum perfluorohexane sulfonate (PFHxS) (Excel Table S1). Top CpGs by *p*-value (FDR < 0.05) in adjusted robust linear regression models for maternal serum perfluorooctane sulfonate (PFOS) (Excel Table S2). Top CpGs by *p*-value (FDR < 0.05) in adjusted robust linear regression models for maternal serum perfluorooctanoate (PFOA) (Excel Table S3). Top CpGs by *p*-value (FDR < 0.05) in adjusted robust linear regression models for maternal serum perfluorononanoate (PFNA) (Excel Table S4). Top CpGs by *p*-value (FDR < 0.05) in adjusted quantile g-compuation for maternal serum per- and polyfluoroalkyl substance (PFAS) mixture and partial weights for individual PFAS (Excel Table S5). Differentially methylated regions (DMRs) associated with maternal serum perfluorohexane sulfonate (PFHxS) (adjusted *p* < 0.05) (Excel Table S6). Differentially methylated regions (DMRs) associated with maternal serum perfluorooctane sulfonate (PFOS) (adjusted *p* < 0.05) (Excel Table S7). Differentially methylated regions (DMRs) associated with maternal serum perfluorooctanoate (PFOA) (adjusted *p* < 0.05) (Excel Table S8). Differentially methylated regions (DMRs) associated with maternal serum perfluorononanoate (PFNA) (adjusted *p* < 0.05) (Excel Table S9). Differentially methylated regions (DMRs) associated with maternal serum per- and polyfluoroalkyl substances (PFAS mixture) (adjusted *p* < 0.05) (Excel Table S10). Results of robust linear regression models (raw *p* < 0.05) for the association between methylation in maternal serum at 132 CpGs associated with maternal serum PFAS and its mixture, and reduced birth size (Excel Table S11). Top CpGs in adjusted robust linear regression with interaction term on infant sex (FDR < 0.05) for maternal serum perfluorohexane sulfonate (PFHxS) (Excel Table S12). Top CpGs in adjusted robust linear regression with interaction term on infant sex (FDR < 0.05) for maternal serum perfluorooctane sulfonate (PFOS) (Excel Table S3). Top CpGs in adjusted robust linear regression with interaction term on infant sex (FDR < 0.05) for maternal serum perfluorooctanoate (PFOA) (Excel Table S14). Top CpGs in adjusted robust linear regression with interaction term on infant sex (FDR < 0.05) for maternal serum perfluorononanoate (PFNA) (Excel Table S15). Top CpGs in adjusted robust linear regression with interaction term on infant sex (FDR < 0.05) and main effect of PFAS (FDR < 0.05) in the Atlanta African American Maternal-Child Cohort, 2014–2020 (Excel Table S16) (XLSX)
Table S1 for total effect population characteristics in this cohort (*N* = 510). Table S2 for the distribution of maternal serum PFAS in the analysis subset and the total effect. Table S3 for Spearman correlation coefficients of natural log-transformed maternal serum PFAS concentrations in the analysis subset and total effect. Table S4 for the number of significant CpG sites based on different cut-offs. Table S5 for mediation analysis of CpGs on the association of PFAS with birth size indicators. Table S6 for CpGs previously reported to be associated with PFAS in birth cohorts. Table S7 for CpGs previously reported to be associated with birth outcomes in birth cohorts. Figure S1 for participant flow diagram. Figure S2 for DAG shows the association of PFAS with (A) birth size indicators and (B) DNA methylation levels. Figure S3 for total effect of PFAS and birth size indicators. Figure S4 for the analytical subsets on the total effect of PFAS and birth size indicators. Figure S5 for PCA on PFAS-associated DMRs with birth size indicators. Figure S6 for scatter plots for the effects of PFAS on DNA methylation levels by infant sex (PDF)
