# Supplementary material for: Maternal Epigenetic Signatures Linking Prenatal Serum PFAS Concentrations to Birth Size in an African American Maternal–Child Cohort
Source: Environ Sci Technol. 2026 Sep 23;60(39):27579–94. doi: 10.1021/acs.est.6c03113 (PMC13648241; doi:10.1021/acs.est.6c03113)
Supplement: Supplementary file 1 [file es6c03113_si_002.pdf]

Supporting Information

Title: Maternal Epigenetic Signatures Linking Prenatal Serum PFAS Concentrations to Birth Size in an African American Maternal–Child Cohort

Authors: Youran Tan[1], Anne L. Dunlop[2], Todd M. Everson[1], Alicia K Smith[2], Stephanie M. Eick[1], Ziyin Tang[1], Shudi Pan[1], Neha Sehgal[1], Dana Boyd Barr[1], Kurunthachalam Kannan[3], P. Barry Ryan[1], Carmen J. Marsit[1], Donghai Liang[1*]

Affiliations:
[1]Gangarosa Department of Environmental Health, Rollins School of Public Health, Emory University, Atlanta, GA, 30322, USA
[2]Department of Gynecology and Obstetrics, School of Medicine, Emory University, Atlanta, GA, 30322, USA
[3]Wadsworth Center, New York State Department of Health, Albany, NY, 12237, USA

*Corresponding Author

Donghai Liang, PhD

Associate Professor, Gangarosa Department of Environmental Health

Emory University Rollins School of Public Health

1518 Clifton Rd

Atlanta, GA 30329, USA

donghai.liang@emory.edu

404-712-9583

- Number of pages- 15
- Number of tables- 7
- Number of figures- 6

Table S1. Sociodemographic and clinical characteristics of pregnant women or total effect in the Atlanta African American Maternal-Child Cohort, 2014-2020 (N=510).

	N=510	N=296	P-value *
**Maternal Age**			
Mean (SD)	25.0 (4.91)	25.0 (4.67)	1.00
**Maternal Early Pregnancy BMI (kg/m^2)**			
Mean (SD)	28.6 (7.77)	28.4 (7.65)	0.72
**Gestational Age at Delivery (weeks)**			
Mean (SD)	38.5 (2.43)	38.4 (2.40)	0.57
**Birth Weight (grams)**			
Mean (SD)	3040 (597)	3030 (585)	0.82
**Birth Weight Z-score for Gestational Age**			
Mean (SD)	-0.45 (1.05)	-0.45 (1.07)	1.00
	**N (%)**	**N (%)**	
**Marital Status**			
Married or cohabiting	242 (47 %)	143 (48 %)	0.81
Not Married or cohabiting	268 (53 %)	153 (52 %)	
**Maternal Education**			
< High school education	81 (16 %)	48 (16 %)	0.86
High School or Some College	196 (38 %)	116 (39 %)	
College Degree	149 (29 %)	90 (30 %)	
Graduate Degree	84 (16 %)	42 (14 %)	
**Income to Poverty Ratio**			
<100%	224 (44 %)	145 (49 %)	0.54
100-150%	116 (23 %)	63 (21 %)	
150-300%	105 (21 %)	52 (18 %)	
>300%	65 (13 %)	36 (12 %)	
**Delivery Hospital**			
Emory	206 (40%)	107 (36 %)	0.23
Grady	304 (60%)	189 (64 %)	
**Parity**			
0	236 (46 %)	132 (45 %)	0.64
1+	274 (54 %)	164 (55 %)	
**Tobacco Use prior to pregnancy**			
No	421 (83 %)	240 (81 %)	0.60
Yes	89 (17 %)	56 (19 %)	
**Marijuana Use prior to pregnancy**			
No	338 (66 %)	188 (64 %)	0.43
Yes	172 (34 %)	108 (36 %)	
**Alcohol Use prior to pregnancy**			
No	447 (88 %)	265 (90 %)	0.42
Yes	63 (12 %)	31 (10 %)	

**Infant Sex**			
Male	250 (49 %)	143 (48 %)	0.85
Female	260 (51 %)	153 (52%)	
**LBW (<2,500 g)**			
No	445 (87 %)	258 (87 %)	0.97
Yes	65 (13%)	38 (13 %)	
**SGA (<10th percentiles)**			
No	399 (78 %)	230 (78 %)	0.86
Yes	111 (22 %)	66 (22 %)	

Abbreviations: SD: standard deviation; BMI: body mass index; LBW: low birth weight; SGA: small for gestational age.
[*] P-values compare the full analytic cohort with available PFAS and birth outcome data (N = 510) and the methylation subset (N = 296). Welch's t-test was used for continuous variables, and Pearson's chi-square test was used for categorical variables.

Table S2. Distribution of maternal serum per-and polyfluoroalkyl substances (PFAS) in analysis subset and total effect in Atlanta African American Maternal-Child cohort, 2014-2020.

				Percentiles				
	**%> LOD**	**LOD[*]**	**GM (GSD)**	**5%**	**25%**	**50%**	**75%**	**100%**
**PFAS (ng/mL) (N=296)**								
PFHxS	96.28	0.02/0.20	0.94 (1.94)	0.28	0.70	1.03	1.46	4.28
PFOS	98.99	0.02/0.05	1.87 (2.07)	0.55	1.38	2.00	2.99	12.42
PFOA	97.64	0.35/0.20	0.62 (2.28)	0.12	0.46	0.71	1.06	3.04
PFNA	96.28	0.02/0.10	0.22 (2.36)	0.04	0.15	0.27	0.40	2.27
**PFAS (ng/mL) (N=510)**								
PFHxS	97.65	0.02/0.20	1.18 (2.04)	0.32	0.82	1.24	1.77	6.17
PFOS	98.24	0.02/0.05	1.90 (2.43)	0.54	1.38	2.14	3.22	12.42
PFOA	97.65	0.35/0.20	0.62 (2.31)	0.12	0.45	0.70	1.06	4.42
PFNA	97.06	0.02/0.10	0.26 (2.34)	0.05	0.17	0.29	0.47	2.27

Abbreviations: LOD: limit of detection; GM: geometric mean; GSD: geometric standard deviation; PFHxS: perfluorohexane sulfonic acid; PFOS: perfluorooctane sulfonic acid; PFOA: perfluorooctanoic acid; PFNA, perfluorononanoic acid.
* Multiple limits of detection (LOD) are provided when PFAS were measured at two different labs.

Table S3. Spearman correlation coefficients of natural log-transformed maternal serum PFAS concentrations in Atlanta African American Maternal-Child cohort, 2014-2020.

**N=296**	**PFHxS**	**PFOS**	**PFOA**	**PFNA**	**N=510**	**PFHxS**	**PFOS**	**PFOA**	**PFNA**
**PFHxS**	1	0.66*	0.48*	0.35*	**PFHxS**	1	0.45*	0.34*	0.43*
**PFOS**		1	0.64*	0.63*	**PFOS**		1	0.61*	0.55*
**PFOA**			1	0.76*	**PFOA**			1	0.66*
**PFNA**				1	**PFNA**				1

Note: The significant correlation was marked by * (p<0.05).
Abbreviations: PFHxS: perfluorohexane sulfonic acid; PFOS: perfluorooctane sulfonic acid; PFOA: perfluorooctanoic acid; PFNA: perfluorononanoic acid.

Table S4. The number of significant CpG sites based on different cut-offs using Bonferroni and False Discovery Rate (FDR) correction.

	FDR q < 0.05	Bonferroni q < 0.05
PFHxS	34	4
PFOS	60	7
PFOA	32	4
PFNA	10	1
Mixture Targeted[*]	98	17

Note: Only for significant CpG sites (FDR<0.05) from individual models (number of unique CpG sites=132)
Abbreviations: PFHxS: perfluorohexane sulfonic acid; PFOS: perfluorooctane sulfonic acid; PFOA: perfluorooctanoic acid; PFNA: perfluorononanoic acid.

Table S5. Mediation analysis of CpGs on the association of PFAS with birth size indicators (P-value <0.10).

**CpG**	**Associated PFAS**	**Outcome**	**Total Effect**	**ACME**	**Proportion of mediation**	**P-value**
cg24626310 (*ABHD12*)	PFOS	BWZ	0.002 (-0.078, 0.093)	0.017 (0.003, 0.038)	8.21%	0.020
		SGA	0.001 (-0.048, 0.024)	-0.009 (-0.019, -0.002)	-14.74%	0.006
cg26859549 (*CTIF*)	PFOS	BWZ	-0.001 (-0.081, 0.100)	-0.016 (-0.035, -0.003)	12.60%	0.016
cg08933467 (*FCRL1*)	PFOA	BWZ	-0.077 (-0.346, 0.147)	-0.031 (-0.085, 0.003)	0.40%	0.062
		SGA	0.033 (-0.083, 0.112)	0.010 (0.001, 0.030)	0.31%	0.040
cg08573893 (*WDYHV1*)	PFHxS	SGA	0.029 (-0.036, 0.084)	-0.012 (-0.029, -0.001)	-0.40%	0.022

Note: P-value refers to the p-value of the estimate of average causal mediation effects (ACME). P-values between 0.05 and 0.10 were considered as borderline significant. The proportion of mediation was calculated by ACME/ADE (average direct effects (ADE) + ACME)
Abbreviations: ACME: average causal mediation effects; SGA: small for gestational age; BWZ: birth weight z-score for gestational age; PFHxS: perfluorohexane sulfonate; PFOA: perfluorooctanoate; PFOS: perfluorooctane sulfonate.

Table S6. CpGs previously reported to be associated with per- and polyfluoroalkyl substances (PFAS) in birth cohorts and identified in the present study.

CpG	Chr	Position	Gene annotation	Relation to gene	Exposure in current study	Associated indicators in previous study	Biomatrix for study sample	Authors
cg16375198	chr7	2354210	*SNX8*	TSS200	PFOA (-)	PFDA (-)	Cord blood	Starling et al., 2020
cg26163375	chr1	1209720	*UBE2J2*	TSS1500	PFHxS (-)	PFDA (-)		
cg04025879	chr22	43573735	*TTLL12*	Body	PFOA (+)	PFNA (-)		
cg18101225	chr4	15964913	*FGFBP2*	TSS200	PFOA (-)	PFHxS (-)		
cg12138678	chr13	43566618	*EPSTI1*	TSS1500	PFOA (-)	PFOS (-); PFNA (-); PFDA (-)		
cg05730996	chr16	11351330	*SOCS1*	TSS1500	PFOA (-), PFAS mixture (-)	PFOS (-)		
cg06274117	chr2	99438242	*C2orf55*	Body	PFOS (+), PFAS mixture (+)	PFOS (+); PFHxS (-)		
cg18746719	chr11	100092054	*CNTN5*	Body	PFOS (-), PFAS mixture (-)	PFOS (+); PFDA (+)		
cg18322765	chr17	74307529	*PRPSAP1*	3'UTR	PFHxS (+), PFAS mixture (+)	PFOA (-); PFNA (-)		
cg05802141	chr16	29911556	*SEZ6L2*	TSS1500	PFHxS (-)	PFNA (-)	Cord blood; Peripheral leukocytes	Liu et al., 2022
cg18746719	chr11	100092054	*CNTN5*	Body	PFOS (-), PFAS mixture (-)	PFOS (+)		

Note: (+/-) indicates the positive/negative directions of effect
Abbreviations: PFHxS: perfluorohexane sulfonic acid; PFOS: perfluorooctane sulfonic acid; PFOA: perfluorooctanoic acid; PFNA: perfluorononanoic acid.

Table S7. CpGs previously reported to be associated with birth outcomes in birth cohorts and identified in the present study.

CpG	Chr	Position	Gene annotation	Relation to gene	Exposure in current study	Associated indicators in previous study	Biomatrix for study sample	Authors
cg01571001	chr3	128215433			PFHxS (+)	Gestational length (-)	Cord blood	Kashima et al., 2021; Bohlin et al., 2016
cg18101225	chr4	15964913	*FGFBP2*	TSS200	PFOA (-)			
cg00675737	chr16	89462359	*ANKRD11*	5'UTR	PFNA (-), PFAS mixture (-)	Gestational length (-)	Cord blood	Kashima et al., 2021
cg05730996	chr16	11351330	*SOCS1*	TSS1500	PFOA (-), PFAS mixture (-)	Gestational length (-)	Cord blood	Bohlin et al., 2016
cg25485913	chr19	49568962						

Note: (+/-) indicates the positive/negative directions of effect
Abbreviations: PFHxS: perfluorohexane sulfonic acid; PFOA: perfluorooctanoic acid; PFNA: perfluorononanoic acid.

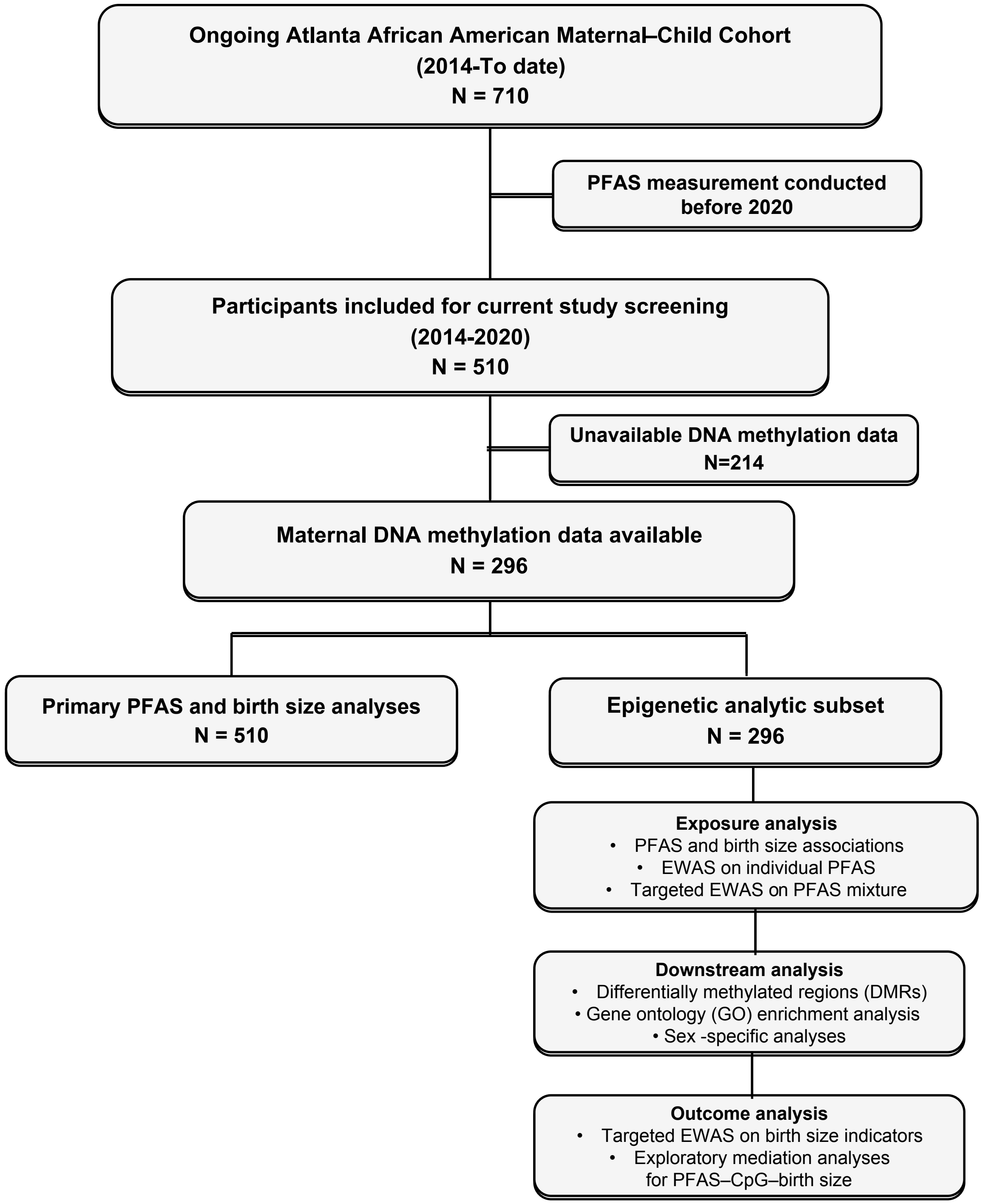

Figure S1. Participant flow diagram for analytic samples.

(A).

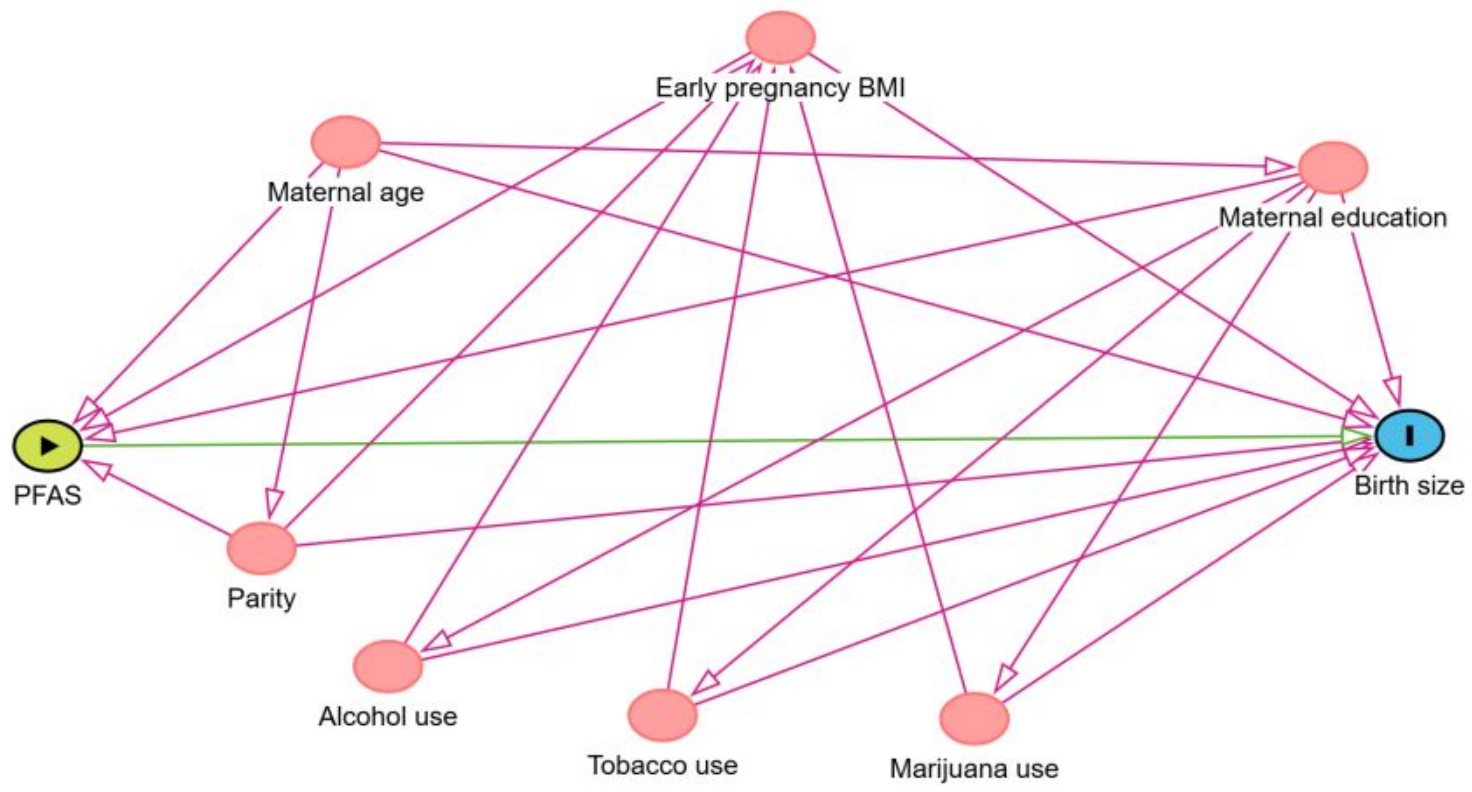

(B).

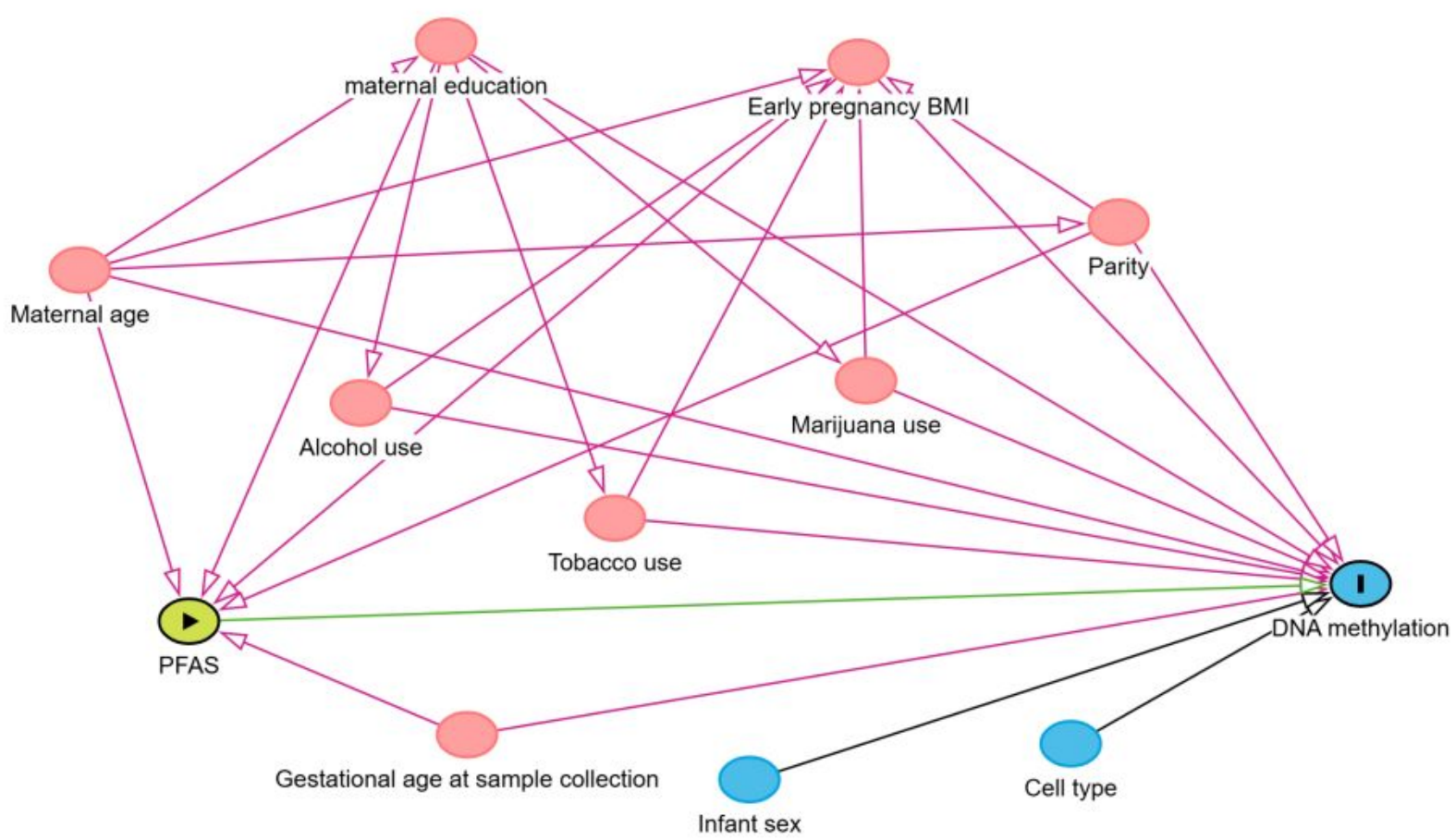

Figure S2. Directed acyclic graph (DAG) shows the association of per-and polyfluoroalkyl substances (PFAS) exposure with (A) birth size indicators and (B) DNA methylation levels.

Note: Green lines represent causal paths, and red lines are biasing paths. Infant's sex is required for estimating the association between PFAS and birth size indicators and not consider as a covariate.

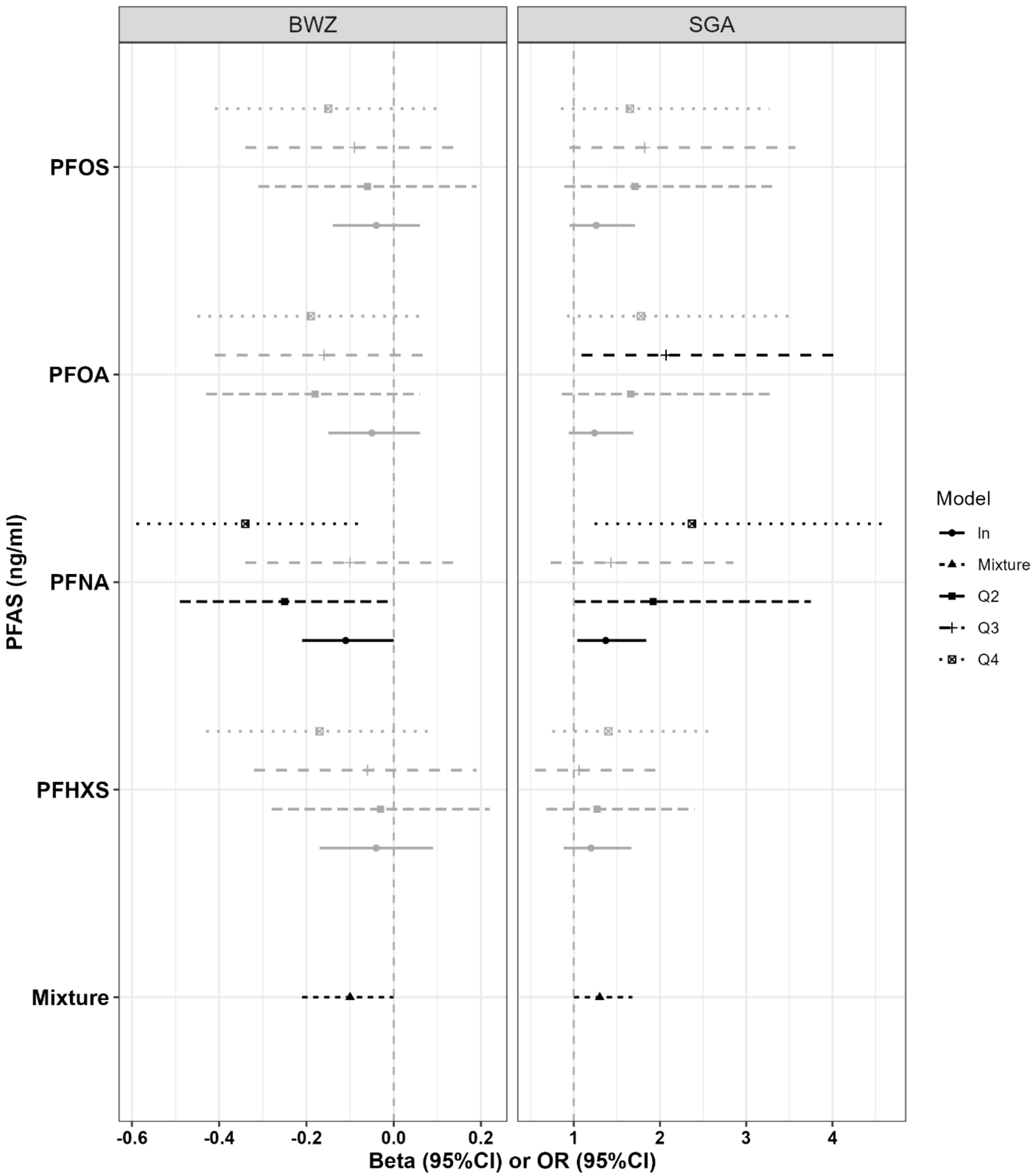

Figure S3. Associations of individual per- and polyfluoroalkyl substances (PFAS) and PFAS mixture with birth weight z-score (BWZ) and small-for-gestational age (SGA) in pregnant African American women in the Atlanta area, 2014-2020 (N=510).

Note: The significant associations ($p < 0.05$) were bold. All models were adjusted for maternal age, maternal education, early pregnancy BMI, substance use and parity.

Abbreviations: PFHxS, perfluorohexane sulfonic acid; PFOS, perfluorooctane sulfonic acid; PFOA, perfluorooctanoic acid; PFNA, perfluorononanoic acid; CI: confidence interval; OR: odds ratio; ln: natural log transform; Q2: quartile 2; Q3: quartile 3; Q4: quartile 4.

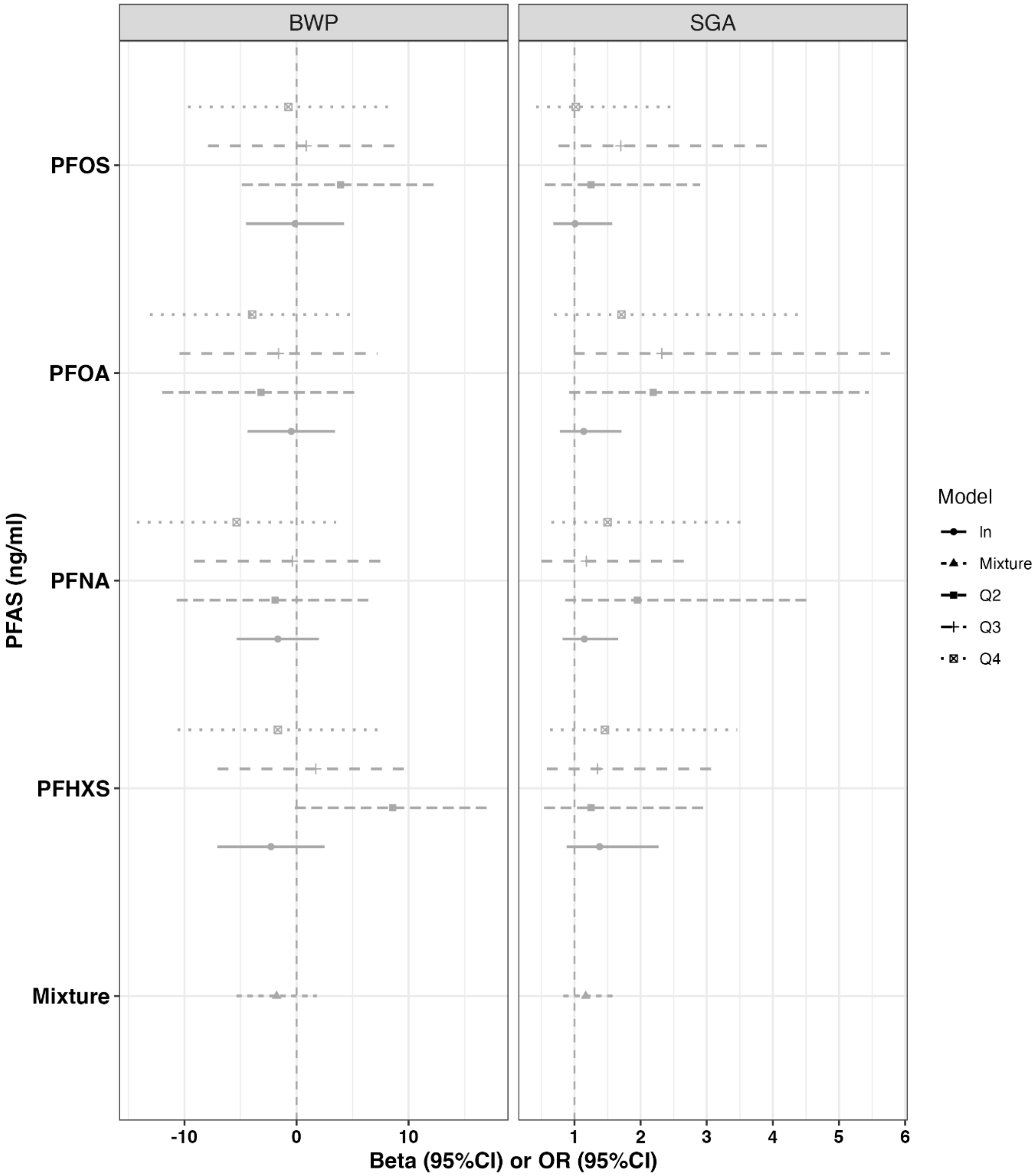

Figure S4. Associations of individual per-and polyfluoroalkyl substances (PFAS) and PFAS mixture with birth weight z-score for gestational age (BWZ) and small-for-gestational age (SGA) in pregnant African American women in the Atlanta area, 2014-2020 (N=296).

Note: All models were adjusted for maternal age, maternal education, early pregnancy BMI, substance use and parity.

Abbreviations: PFHxS: perfluorohexane sulfonic acid; PFOS: perfluorooctane sulfonic acid; PFOA: perfluorooctanoic acid; PFNA: perfluorononanoic acid; CI: confidence interval; OR: odds ratio; ln: natural log transform; Q2: quartile 2; Q3: quartile 3; Q4: quartile 4.

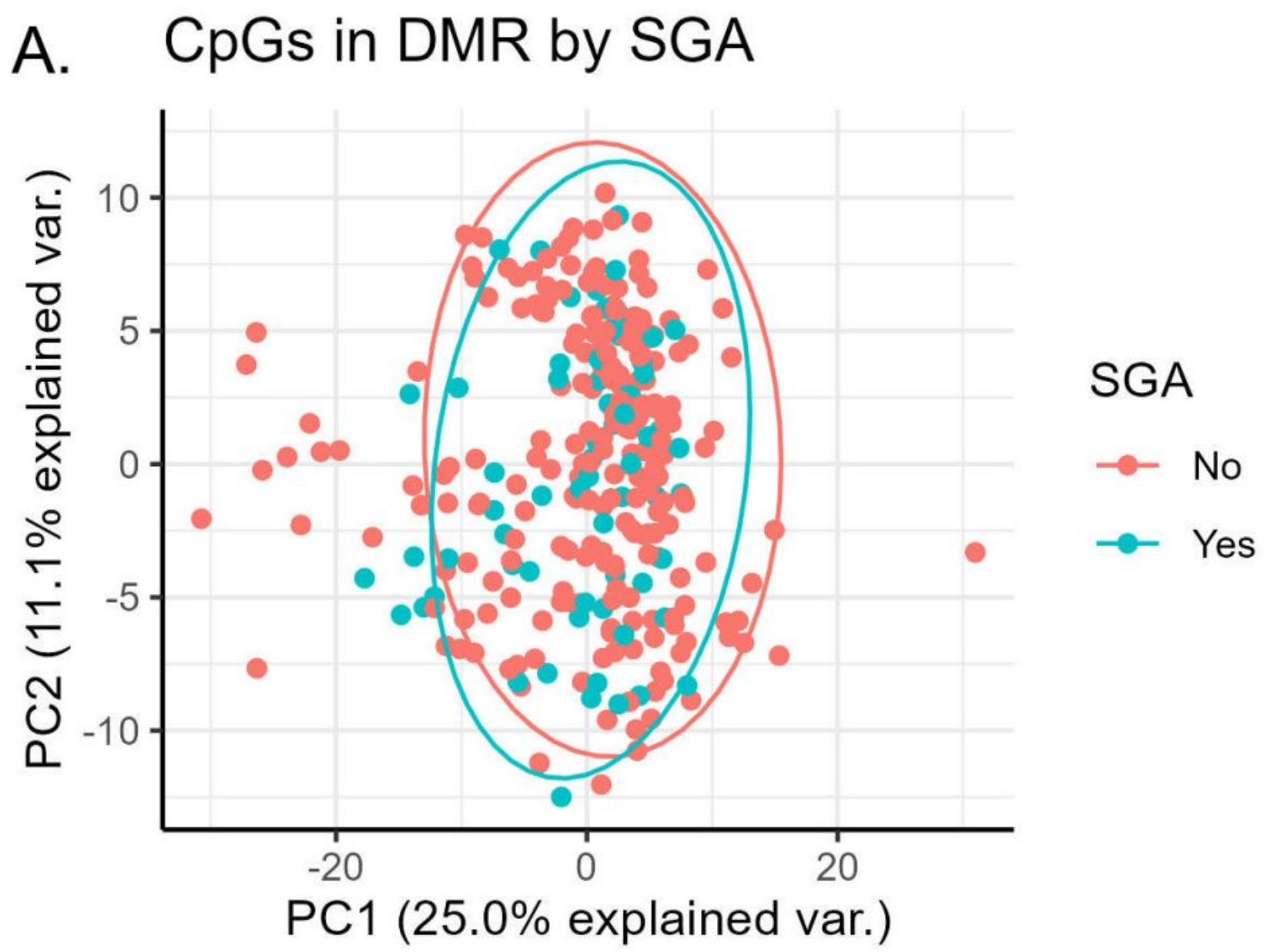

B.

Outcome	N	Beta (95% CI)
SGA	296	-0.5 (-2.51, 1.52)
BWZ	296	0.22 (-0.58, 1.02)

Figure S5. Principal components analysis (PCA) on per-and polyfluoroalkyl substances (PFAS) associated differentially methylated regions (DMRs) with birth size indicators. (A) PCA by small for gestational age (SGA); (B) Linear regression for the association between PC1 and birth size indicators.
Abbreviations: PC: principal components; Var: variation; CI: confidence interval.

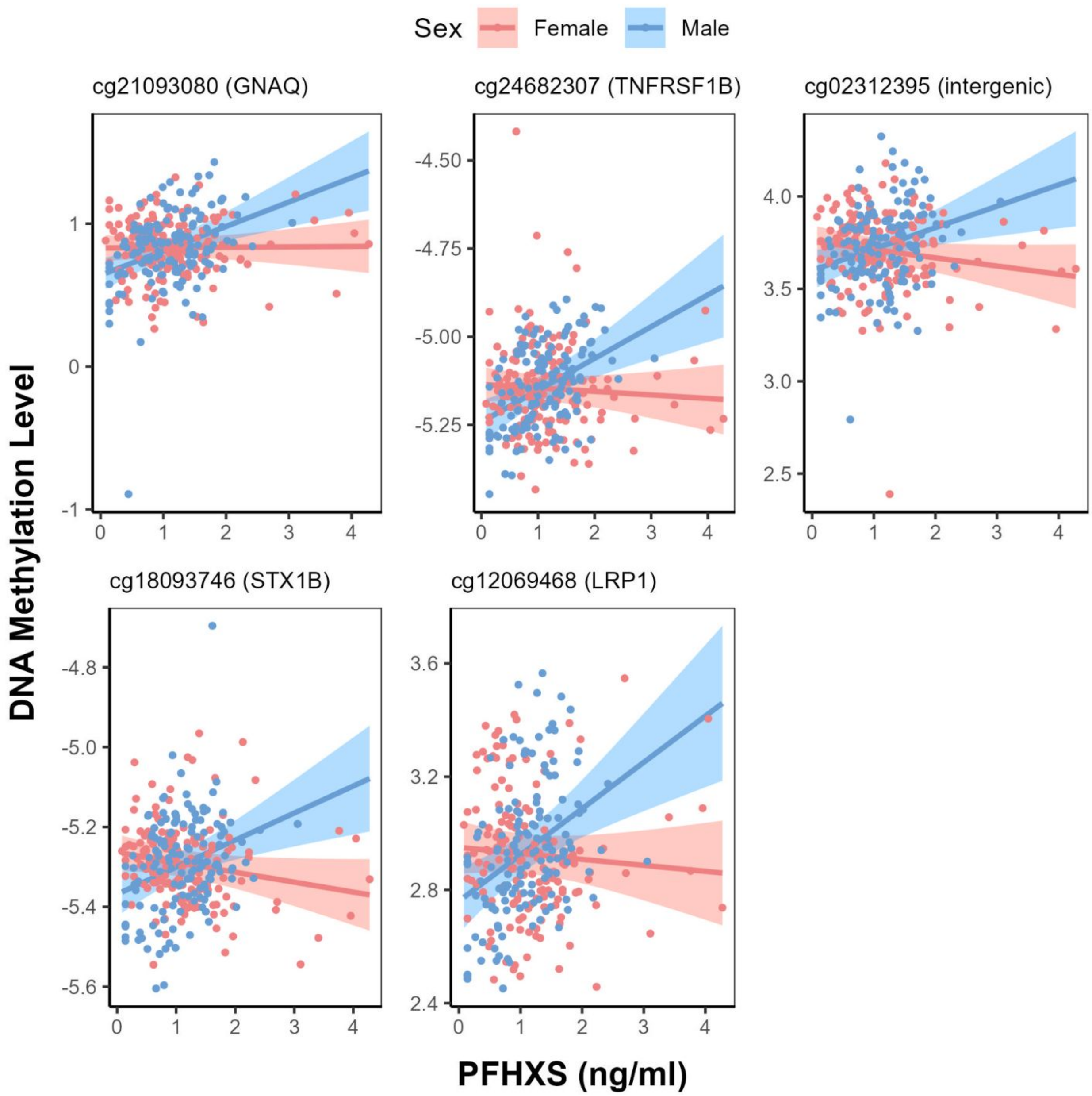

Sex
Female
Male
cg21093080 (GNAQ)
cg24682307 (TNFRSF1B)
cg02312395 (intergenic)
cg18093746 (STX1B)
cg12069468 (LRP1)
DNA Methylation Level
PFHXS (ng/ml)

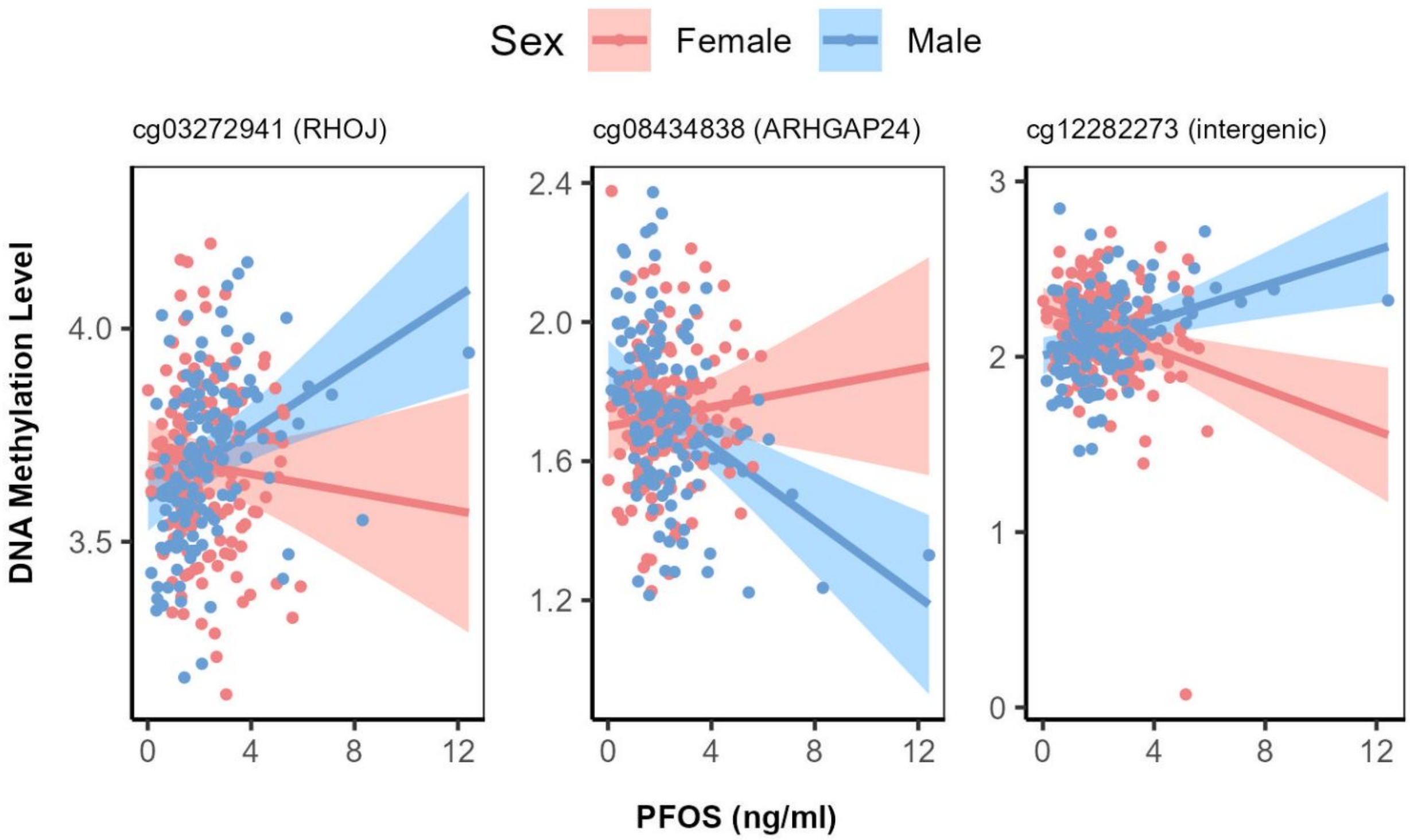

Sex
Female
Male
cg03272941 (RHOJ)
cg08434838 (ARHGAP24)
cg12282273 (intergenic)
DNA Methylation Level
PFOS (ng/ml)

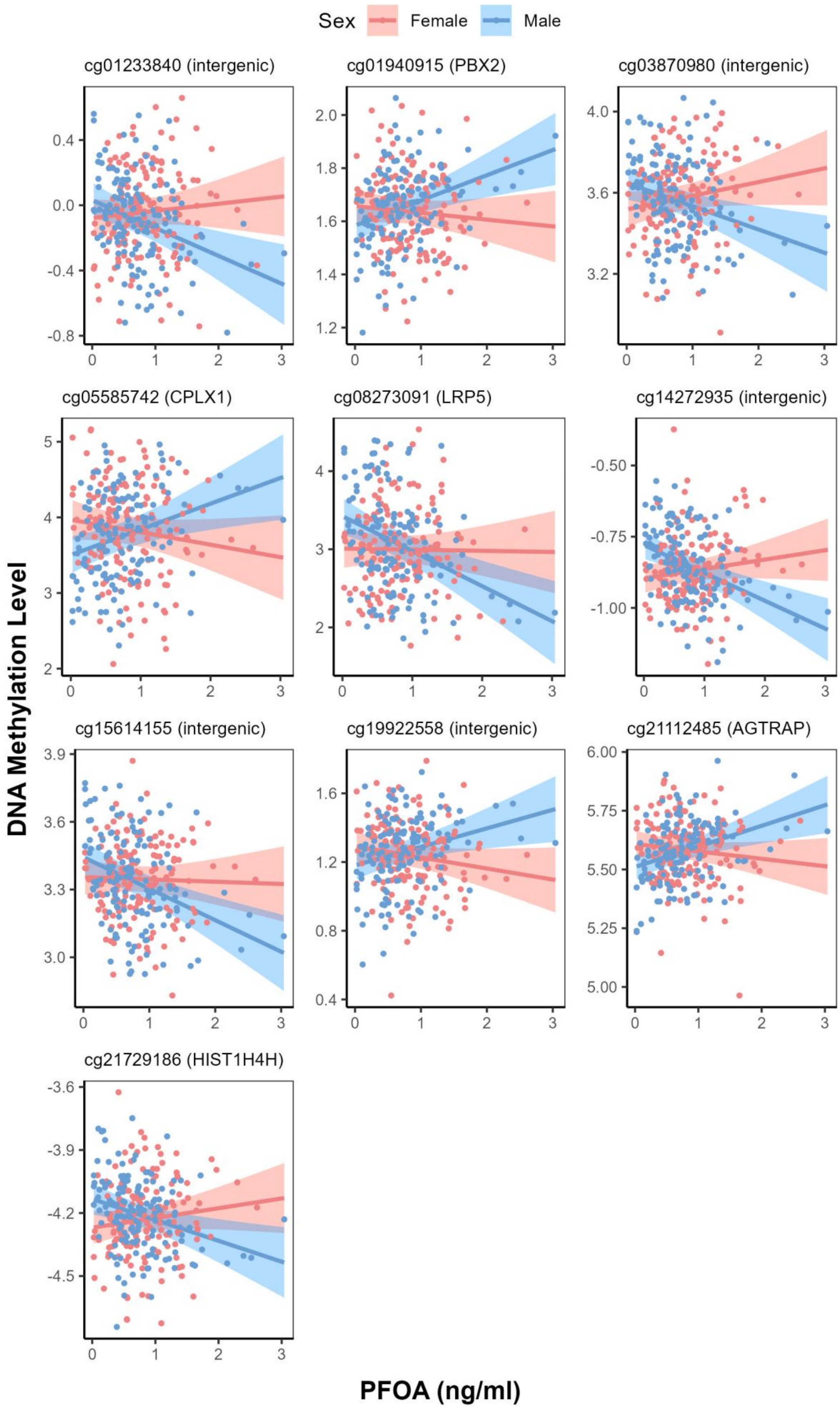
Sex
Female
Male
cg01233840 (intergenic)
cg01940915 (PBX2)
cg03870980 (intergenic)
cg05585742 (CPLX1)
cg08273091 (LRP5)
cg14272935 (intergenic)
cg15614155 (intergenic)
cg19922558 (intergenic)
cg21112485 (AGTRAP)
cg21729186 (HIST1H4H)
DNA Methylation Level
PFOA (ng/ml)

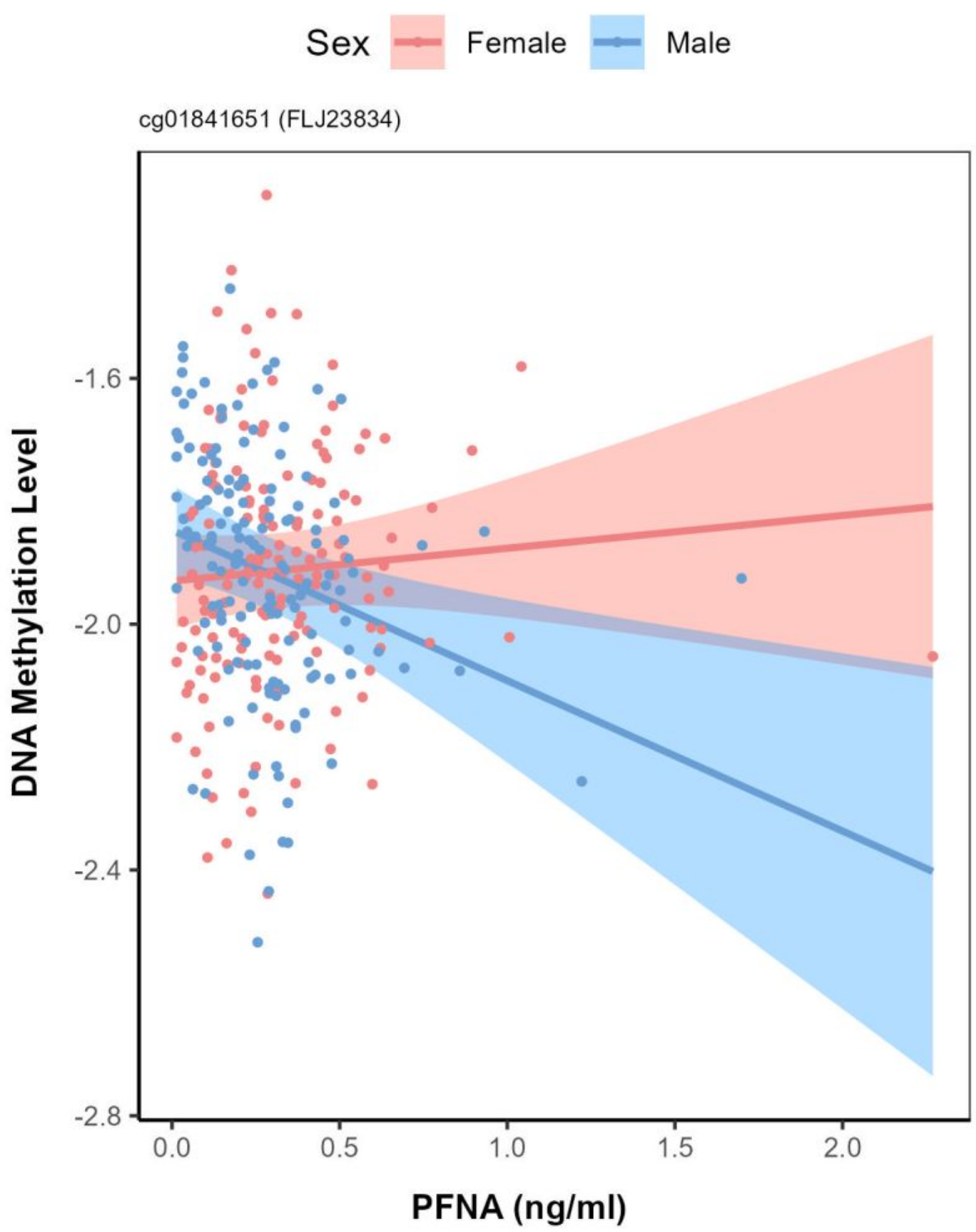

Figure S6. Scatter plots for the effects of per-and polyfluoroalkyl substances (PFAS) on DNA methylation levels by infant sex, holding all other variables constant.

Note: All models were adjusted for maternal age, infant sex, maternal education, early pregnancy BMI, substance use, parity, gestational age at sample collection, cell types and surrogate variables. Only CpGs where the effect estimates of PFAS and interaction term in individual PFAS-CpGs models were significant (FDR q < 0.05) were shown.

Abbreviations: PFHxS: perfluorohexane sulfonic acid; PFOS: perfluorooctane sulfonic acid; PFOA: perfluorooctanoic acid; PFNA: perfluorononanoic acid.

Supplementary Excel Table S1. Top CpGs by p-value (FDR<0.05) in adjusted robust linear regression models for maternal serum perfluorohexane sulfonate (PFHxS).
Supplementary Excel Table S2. Top CpGs by p-value (FDR<0.05) in adjusted robust linear regression models for maternal serum perfluorooctane sulfonate (PFOS).
Supplementary Excel Table S3. Top CpGs by p-value (FDR<0.05) in adjusted robust linear regression models for maternal serum perfluorooctanoate (PFOA).
Supplementary Excel Table S4. Top CpGs by p-value (FDR<0.05) in adjusted robust linear regression models for maternal serum perfluorononanoate (PFNA).
Supplementary Excel Table S5. Top CpGs by p-value (FDR<0.05) in adjusted quantile g-compuation for maternal serum per- and polyfluoroalkyl substance (PFAS) mixture and partial weights for individual PFAS.
Supplementary Excel Table S6. Differentially methylated regions (DMRs) associated with maternal serum perfluorohexane sulfonate (PFHxS) (adjusted p <0.05).
Supplementary Excel Table S7. Differentially methylated regions (DMRs) associated with maternal serum perfluorooctane sulfonate (PFOS)(adjusted p <0.05).
Supplementary Excel Table S8. Differentially methylated regions (DMRs) associated with maternal serum perfluorooctanoate (PFOA) (adjusted p <0.05).
Supplementary Excel Table S9. Differentially methylated regions (DMRs) associated with maternal serum perfluorononanoate (PFNA) (adjusted p <0.05).
Supplementary Excel Table S10. Differentially methylated regions (DMRs) associated with maternal serum per- and polyfluoroalkyl substances (PFAS mixture) (adjusted p <0.05).
Supplementary Excel Table S11. Results of robust linear regression models (raw p<0.05) for the association between methylation in maternal serum at 132 CpGs associated with maternal serum PFAS and its mixture, and reduced birth size.
Supplementary Excel Table S12. Top CpGs in adjusted robust linear regression with interaction term on infant sex (FDR<0.05) for maternal serum perfluorohexane sulfonate (PFHxS).
Supplementary Excel Table S3. Top CpGs in adjusted robust linear regression with interaction term on infant sex (FDR<0.05) for maternal serum perfluorooctane sulfonate (PFOS).
Supplementary Excel Table S14. Top CpGs in adjusted robust linear regression with interaction term on infant sex (FDR<0.05) for maternal serum perfluorooctanoate (PFOA).
Supplementary Excel Table S15. Top CpGs in adjusted robust linear regression with interaction term on infant sex (FDR<0.05) for maternal serum perfluorononanoate (PFNA).
Supplementary Excel Table S16. Top CpGs in adjusted robust linear regression with interaction term on infant sex (FDR<0.05) and main effect of PFAS (FDR <0.05) in the Atlanta African American Maternal-Child Cohort, 2014-2020.
